# Burden of Six Healthcare-Associated Infections on European Population Health: Estimating Incidence-Based Disability-Adjusted Life Years through a Population Prevalence-Based Modelling Study

**DOI:** 10.1371/journal.pmed.1002150

**Published:** 2016-10-18

**Authors:** Alessandro Cassini, Diamantis Plachouras, Tim Eckmanns, Muna Abu Sin, Hans-Peter Blank, Tanja Ducomble, Sebastian Haller, Thomas Harder, Anja Klingeberg, Madlen Sixtensson, Edward Velasco, Bettina Weiß, Piotr Kramarz, Dominique L. Monnet, Mirjam E. Kretzschmar, Carl Suetens

**Affiliations:** 1 European Centre for Disease Prevention and Control, Stockholm, Sweden; 2 Julius Center for Health Sciences and Primary Care, University Medical Center Utrecht, Utrecht, The Netherlands; 3 Robert Koch Institute, Berlin, Germany; 4 Centre for Infectious Disease Control, National Institute for Public Health and the Environment, Bilthoven, The Netherlands; University of Geneva Hospitals and Medical School, SWITZERLAND

## Abstract

**Background:**

Estimating the burden of healthcare-associated infections (HAIs) compared to other communicable diseases is an ongoing challenge given the need for good quality data on the incidence of these infections and the involved comorbidities. Based on the methodology of the Burden of Communicable Diseases in Europe (BCoDE) project and 2011–2012 data from the European Centre for Disease Prevention and Control (ECDC) point prevalence survey (PPS) of HAIs and antimicrobial use in European acute care hospitals, we estimated the burden of six common HAIs.

**Methods and Findings:**

The included HAIs were healthcare-associated pneumonia (HAP), healthcare-associated urinary tract infection (HA UTI), surgical site infection (SSI), healthcare-associated *Clostridium difficile* infection (HA CDI), healthcare-associated neonatal sepsis, and healthcare-associated primary bloodstream infection (HA primary BSI). The burden of these HAIs was measured in disability-adjusted life years (DALYs). Evidence relating to the disease progression pathway of each type of HAI was collected through systematic literature reviews, in order to estimate the risks attributable to HAIs. For each of the six HAIs, gender and age group prevalence from the ECDC PPS was converted into incidence rates by applying the Rhame and Sudderth formula. We adjusted for reduced life expectancy within the hospital population using three severity groups based on McCabe score data from the ECDC PPS. We estimated that 2,609,911 new cases of HAI occur every year in the European Union and European Economic Area (EU/EEA). The cumulative burden of the six HAIs was estimated at 501 DALYs per 100,000 general population each year in EU/EEA. HAP and HA primary BSI were associated with the highest burden and represented more than 60% of the total burden, with 169 and 145 DALYs per 100,000 total population, respectively. HA UTI, SSI, HA CDI, and HA primary BSI ranked as the third to sixth syndromes in terms of burden of disease. HAP and HA primary BSI were associated with the highest burden because of their high severity. The cumulative burden of the six HAIs was higher than the total burden of all other 32 communicable diseases included in the BCoDE 2009–2013 study. The main limitations of the study are the variability in the parameter estimates, in particular the disease models’ case fatalities, and the use of the Rhame and Sudderth formula for estimating incident number of cases from prevalence data.

**Conclusions:**

We estimated the EU/EEA burden of HAIs in DALYs in 2011–2012 using a transparent and evidence-based approach that allows for combining estimates of morbidity and of mortality in order to compare with other diseases and to inform a comprehensive ranking suitable for prioritization. Our results highlight the high burden of HAIs and the need for increased efforts for their prevention and control. Furthermore, our model should allow for estimations of the potential benefit of preventive measures on the burden of HAIs in the EU/EEA.

## Abstract 2

In a population prevalence-based modelling study, Alessandro Cassini and colleagues estimate the burden of six healthcare-associated infections on European population health in disability-adjusted life years.

## Author Summary

**Why Was This Study Done?:**

- Infections acquired in hospitals are a common and largely preventable complication of hospitalisation and surgery affecting approximately 1 in 20 patients.
- We measured the burden of healthcare-associated infections (HAIs) in disability-adjusted life years (DALYs), thus also enabling comparison with the burden of other diseases.
- Calculating the burden of HAIs is challenging given the underlying disease that caused the initial hospitalisation.

**What Did the Researchers Do and Find?:**

- We estimated the burden of six common HAIs in DALYs: healthcare-associated pneumonia (HAP), healthcare-associated urinary tract infection (HA UTI), surgical site infection (SSI), healthcare-associated *C*. *difficile* infection (HA CDI), healthcare-associated neonatal sepsis, and healthcare-associated primary bloodstream infection (HA primary BSI).
- The data source for the number of infections was the European point prevalence survey of healthcare-associated infections and antimicrobial consumption in acute care hospitals.
- HAI-attributable outcomes and length of stay were based on a systematic review of the literature. Moreover, life expectancy was adjusted according to the severity of the underlying condition.
- More than 2.5 million cases of HAI occur in the European Union and European Economic Area (EU/EEA) each year, corresponding to approximately 2.5 million DALYs.
- HAP and HA primary BSI were responsible for the largest part of the total burden of HAIs.
- The total burden of the six HAIs in Europe was higher than that of all other communicable diseases under surveillance at ECDC.

**What Do These Findings Mean?:**

- HAIs represent a significant burden among infectious diseases in Europe, exceeding the burden of other infections like influenza and tuberculosis.
- Increasing efforts for prevention are imperative to decrease this burden.

## Introduction

Healthcare-associated infections (HAIs) are associated with increased morbidity and mortality and excess costs, and because a significant proportion of them are preventable, they are considered to be a marker of quality of patient care [1]. Many studies have attempted to estimate the number of cases of HAIs and of deaths attributable to these infections [2–6]. Such studies have used descriptive methods, modelling approaches, or a combination of the two.

There is a well-established methodology for estimating the burden of diseases that takes into account not only the incidence of the disease but also disabilities associated with their complications and the years of life lost, resulting in a composite health measure, the disability-adjusted life year (DALY) [7]. However, this methodology has not been applied to estimate an overall burden of HAIs. This prevents comparisons of the burden of HAIs to that of other infectious and noninfectious diseases, which would be particularly useful for healthcare professionals, policy makers, and the public.

One of the challenges in the estimation of the burden of HAIs is their special nature. Patients with an HAI are or have recently been hospitalised or were subject to a surgical intervention and have comorbidities that, beside the HAI, also contribute to morbidity and mortality. For this reason, it is essential to study patient outcomes that are specifically attributable to the HAI and not to the underlying disease. This includes calculating the expected individual life years at the age of death for patients with HAI. Moreover, administrative hospital discharge data that are commonly used to estimate the burden of other diseases do not accurately reflect the burden of HAIs, making it necessary to identify other sources of data [8].

We calculated the DALYs aiming at describing the burden of HAIs in acute care hospitals of the European Union and European Economic Area (EU/EEA) using the methodology of the Burden of Communicable Diseases in Europe (BCoDE) project [9,10] and the results of the European Centre for Disease Prevention and Control (ECDC) point prevalence survey (PPS) of HAIs and antimicrobial use in European acute care hospitals [6].

## Methods

### Ethics Statement

This study was based on health information collected and published during the 2011–2012 PPS of HAIs in acute care hospitals within the EU/EEA [6] and did not require informed consent from participants. Reported infectious disease data were provided in aggregate form by specific age and gender strata, without any personal identifiers.

### Study Design

The methodology of the present study was adapted from the BCoDE project [10]. Specifically, the burden of selected HAIs in acute care facilities was expressed through a composite health measure reflecting the burden of disabilities and premature deaths against a prespecified ideal. The approach is incidence-based in order to best express current and future consequences of infections, as well as the effect of future prevention and control interventions.

The present study used a syndrome-based approach and not the pathogen-based approach used for other BCoDE-related outputs, with the exception of *C*. *difficile*. A vast array of pathogens cause HAIs, which can be split according to recognizable and similar syndromes. Moreover, the syndrome approach has more significant public health relevance both in terms of surveillance and in terms of hospital infection prevention and control.

Selection of syndromes was primarily based on availability of incidence data, systematic literature reviews, and discussion within a European panel of experts. The HAIs included in the present study were healthcare-associated urinary tract infection (HA UTI), healthcare-associated primary bloodstream infection (HA primary BSI), healthcare-associated neonatal sepsis, healthcare-associated *C*. *difficile* infection (HA CDI), surgical site infection (SSI), and healthcare-associated pneumonia (HAP), as defined by the EU/EEA case definitions [11].

### Outcome Measure

The DALY is a composite health measure estimating years lived with disabilities (YLDs) following the onset of a disease and of years of life lost due to pre-mature mortality (YLLs) compared to a standardized life expectancy [12]. YLDs include the length of time lived with disabilities (duration) multiplied by disability weights reflecting the ill health incurred. In our study, the latter were derived from the European disability weight project [13–15].

Data on life expectancy were obtained from the Global Burden of Disease 2010 (GBD 2010) standard reference life table with the same life expectancy for males and females, based on the lowest observed death rate for any age group [12].

### Disease Models and Correction for Comorbidities

Since HAIs occur in the context of comorbidities, adjustment for the effect of these comorbidities was necessary. In order to take into account all possible health consequences of HAIs, disease models or outcome trees were developed based on several systematic reviews of the literature, focusing on the attributable risk of complications, death, and length of stay due to the HAI [16,17]. An outcome tree represents the progression pathway of a disease over time, starting with the infection and ending with either recovery, a permanent disability, or death. Health outcomes can include short-term complications (health states within a health outcome) and long-term sequelae. Each health outcome is related to the other outcomes by a transitional probability and includes a duration and a disability weight. The authors critically reviewed each outcome tree stemming from a systematic review of the literature and discussed and agreed on each parameter. The consensus-building procedure entailed four separate stages performed between February and December 2015. The results from the systematic review [17] were reviewed independently by the two authors (AC and DP) and the structure and parameters for the final outcome tree indicatively selected during this first stage. During the second stage, shared views were discussed and their reasons analysed in order to confirm a common rationale. Disagreements were solved by discussion. The third stage included another expert and author (CS), and disagreements were further analysed and discussed until consensus was reached. The final and fourth stage entailed a final review by the head of the HAI programme at ECDC and author of the present study (DM). The final HAI outcome trees were published in the BCoDE toolkit on the ECDC website in December 2015. The disease model parameters are described in detail in the BCoDE toolkit [18] and are available in S1 Models.

Comorbidities also affect the life expectancy of hospitalised patients. Therefore, we categorized the affected hospitalized population according to the McCabe score [19] that was recorded for every patient enrolled in the ECDC PPS. The McCabe score gives an indication of the life expectancy of a patient according to the severity of their underlying disease. Patients are classified in three categories based on whether the underlying disease is nonfatal (McCabe score 1, expected survival of more than 5 y), ultimately fatal (McCabe score 2, expected survival between 1 and 5 y), or rapidly fatal (McCabe score 3, expected survival less than 1 y). The incidence of each HAI was therefore divided into three groups based on McCabe score: McCabe score 1 (standard average life expectancy), McCabe score 2 (3 y average life expectancy), and McCabe score 3 (0.5 y average life expectancy) [19].

SSI incidence and severity vary widely depending on the site and nature of the surgical intervention and the depth of the infection. One way to deal with this variability could have been to focus the systematic review of the literature on SSIs following hip and knee joint replacements and following coronary artery bypass grafts (CABGs), as examples of operations with predominantly acute (CABG) or chronic (joint replacement) infectious complications. However, the results would only partially cover the full range of SSIs. Therefore, as a final decision for the SSI outcome tree, a different approach was chosen: only the acute phase of the disease and the attributable mortality were included based on data on overall SSI outcomes [20].

### Study Population and Incidence

Estimates of the incidence of the selected HAIs were derived from the ECDC PPS, which was conducted in 2011–2012 in 29 EU/EEA Member States and Croatia, and included data from a total of 273,753 patients in 1,149 hospitals [6]. Since only acute care hospitals participated in this ECDC PPS, other healthcare facilities such as long-term care facilities were not included in our study.

The results of the ECDC PPS represent the EU/EEA Member States, with more that 510 million citizens according to 2011 Eurostat data. Five percent of the population was under 5 y old, and 18% was 65 y and over. In the EU/EEA, there were 2,719,634 available beds in hospitals [21], of which 1,840,514 were in acute care with 13,090 discharges of inpatients per 100,000 inhabitants in 2011 [22].

The gender-specific and age-group-specific yearly number of cases of HAIs (further referred to as “patients with HAIs”) was calculated from the rate of new cases of HAI per 100 admissions using the 2011 Eurostat data on the number of inpatient hospital discharges and general population (see S1 Input) [22].

The rate of new cases of each type of HAI per 100 admissions was estimated for each gender, age group, and McCabe score category by converting the stratum-specific prevalence rate from the ECDC PPS into an incidence rate using the Rhame and Sudderth formula, *I* = *P* × *LA*/(*LN* − *INT*). *I* (incidence) is the rate of new patients with HAIs per 100 admissions, *P* (prevalence) is the percentage of patients with HAIs on the day of the PPS, *LA* is the length of stay of all hospitalized patients (irrespective of the presence of an HAI), *LN* is the length of stay of patients with an HAI, and *INT* is the length of stay before the onset of the HAI [23]. *LN—INT*, representing the length of stay of patients with HAIs from HAI onset until discharge, was derived from the median number of days from HAI onset until the day of the PPS. This choice was based on the fact that the average length of stay for all patients (as derived from the hospital data) in the ECDC PPS was best correlated with the median length of stay of patients included on the day of the PPS [6], as patients with a longer stay are overrepresented in any PPS sample. The country-specific average length of stay of all hospitalized patients (LA) was extracted from the ECDC PPS.

Under-reporting is a significant parameter affecting burden of disease estimates [24]. This is also true for the reporting of HAIs in the ECDC PPS, in which a small validation study indicated an average under-reporting factor of 1.25. This validation study was only performed in four EU/EEA Member States and was therefore not deemed indicative of under-reporting of HAIs in the whole EU/EEA. In the present study, we did not apply any correction factor adjusting for underestimation of HAI incidence.

### Computational Analysis and Uncertainty

The final designs of the HAI outcome trees, including their model parameters and uncertainties, were inserted in the BCoDE toolkit [18]. For each type of HAI, three models were generated, and population and life expectancy data were customized to cover all EU/EEA populations according to their McCabe score category. Successively, the gender-specific and age-group-specific yearly numbers of cases of HAIs and uncertainties were inputted relatively to each McCabe score category. Where applicable, inputs for disease model parameters and HAI incidence data included uncertainty intervals, which were incorporated in the calculations as uniform (two variables) or Project Evaluation and Review Techniques (PERT) (three variables) distributions [25]. See S1 Input for detailed age-group- and sex-specific tables inputted in the BCoDE toolkit.

To calculate uncertainty intervals, the BCoDE toolkit models were run at 1,000 iterations of the Monte Carlo simulations with and without a 3.5% annual time discount rate. The option of discounting may allow for possible future comparisons with cost-effectiveness studies on, for example, interventions to prevent HAIs [26].

For each type of HAI, the outputs included the annual number of cases of HAIs, the HAI incidence, the number of deaths attributable to HAIs, and the DALYs per case, as well as the number and the rate per 100,000 population of YLLs, YLDs, and DALYs. For each output, the median and the 95% uncertainty interval (95% UI) based on the input uncertainties were calculated.

## Results

Based on data from 2011–2012, we estimated that 2,609,911 (95% UI: 2,451,235–2,778,451) new cases of HAI occur every year in the EU/EEA. These HAIs accounted for a total of 2,506,091 DALYs (95% UI: 2,163,850–2,877,574) annually in the EU/EEA, corresponding to 501 DALYs per 100,000 general population (95% UI: 429–582). These DALYs consisted of 2 million YLLs (75% of total DALYs) and 681,400 YLDs.

When applying a 3.5% annual time discount rate, HAIs accounted for 1,335,159 DALYs (95% UI: 1,153,291–1,536,343), corresponding to 261 DALYs per 100,000 general population (95% UI: 226–301). The reduction of DALYs when applying time discounting occurred mainly within the McCabe score 1 category.

**Fig 1:**
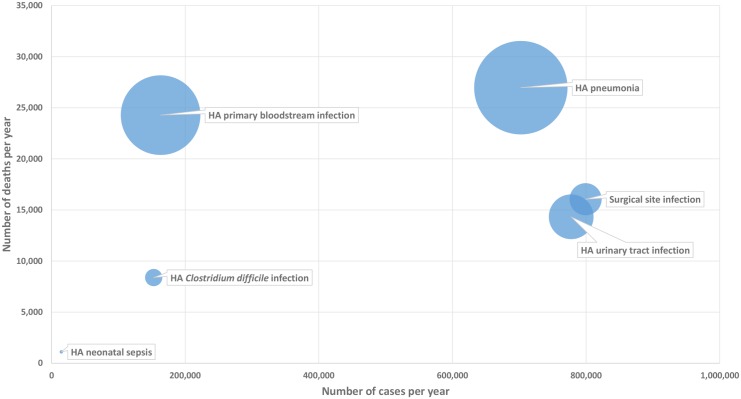
Six healthcare-associated infections according to their number of cases per year (*x*-axis), number of deaths per year (*y*-axis), and DALYs per year (width of bubble), EU/EEA, 2011–2012 (time discounting was not applied). DALY, disability-adjusted life year; HA, healthcare-associated.

For each type of HAI, the relationship between the incidence of HAIs, the number of deaths attributable to HAIs, and the total burden of HAIs in DALYs per 100,000 general population depends on the severity of disease and its long-term complications. This is illustrated by the bubble chart presented in Fig 1.

As shown in this figure, HA primary BSIs, even with a relatively low incidence, generated a high burden of disease due to their high attributable mortality, whereas SSIs that have a higher incidence were associated with a lower burden of disease. More generally, the figure illustrates how the ranking of HAIs may differ depending on which indicator is used for measuring their health burden and therefore gives more detailed information on how different types of HAIs compare in their burden on population health.

More than 60% of the total burden of the six selected HAIs was accounted for by HAP and HA primary BSIs. When only considering the population at risk for HA neonatal sepsis, i.e., newborns (derived from the 2011–2012 average Eurostat number of births in the EU/EEA) instead of the general population, the burden of HA neonatal sepsis rose from 16.8 DALYs per 100,000 general population to 1,592 DALYs per 100,000 newborns. Over 60% of the total DALYs were due to the acute phase of the six HAIs, while the remaining DALYs were due to the sequelae, regardless of time discounting.

The estimates of the burden of the six selected types of HAIs are presented in Table 1. The detailed results for each type of HAI (without time discounting) are shown in S1 Output.

**Table 1:** Estimated annual burden of six healthcare-associated infections, EU/EEA, 2011–2012 (time discounting was not applied).

Healthcare-Associated Infections	Median (95% Uncertainty Interval)							% Total DALYs
	Cases per Year	Incidence (per 100,000 Population)	Deaths per Year	DALYs per Case	YLDs per 100,000 Population	YLLs per 100,000 Population	DALYs per 100,000 Population	
HA Pneumonia	702,315 (664,764–744,419)	138 (130–145)	26,972 (21,859–32,541)	2.2 (1.9–2.4)	67.0 (59.7–74.0)	103 (85.7–121)	169 (149–192)	33.7
HA Primary Bloodstream Infection	163,216 (145,012–182,059)	32 (28.4–35.7)	24,284 (20,824–27,755)	8 (7.2–8.8)	21.2 (17.9–24.9)	123 (104–142)	145 (123–166)	28.9
HA Urinary Tract Infection	777,639 (737,820–820,228)	152 (145–161)	14,334 (11,768–17,162)	0.8 (0.7–0.9)	24.8 (20.8–29.0)	56.4 (47.1–66.5)	81.2 (69.0–94.2)	16.2
Surgical Site Infection	799,185 (762,721–835,448)	156.5 (150–163.7)	16,049 (15,249–16,893)	0.5 (0.5–0.6)	0.8 (0.7–0.8)	57.5 (55.0–59.8)	58.2 (55.7–60.6)	11.6
HA *C*. *difficile* Infection	152,905 (134,053–173,089)	30 (26.3–33.9)	8,382 (6,034–11,152)	1.7 (1.3–2.2)	1.4 (1.1–1.8)	29.8 (22.4–39.6)	31.2 (23.6–41.1)	6.23
HA Neonatal Sepsis	14,651 (7,466–23,873)	2.9 (1.5–4.7)	1,109 (383–2,380)	12.1 (7.6–16.9)	6.9 (3.9–11.0)	9.9 (4.0–18.1)	16.8 (8.9–27.6)	3.35
Overall	2,609,911 (2,451,235–2,778,451)	512 (480–545)	91,130 (76,117–107,883)	25.1 (19.0–31.5)	122 (105–143)	380 (318–447)	501 (429–582)	100

Abbreviations: YLDs, years lived with disability; YLLs, years of life lost due to premature mortality.

**Fig 2:**
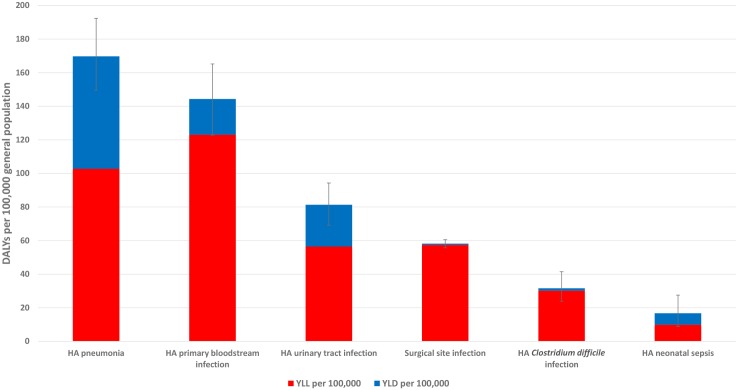
Estimated annual burden of six healthcare-associated infections in DALYs per 100,000 population (median and 95% uncertainty interval), split between YLLs and YLDs, EU/EEA, 2011–2012 (time discounting was not applied).

Fig 2 summarizes the burden of the six types of HAI expressed in annual DALYs per 100,000 general population, distributed between YLLs and YLDs.

A total of 91,130 deaths (95% UI: 76,117–107,883) each year in the EU/EEA were attributable to the six selected HAIs. Fifty-six percent of the estimated attributable deaths were attributable to HAP and HA primary BSIs (Table 1). Sixty-five percent of the deaths attributable to HAIs occurred in patients in the McCabe score 1 category, twenty-five percent in the McCabe score 2 category, and ten percent in the McCabe score 3 category. This distribution was mainly due to the large number of HAIs that occurred in patients with a McCabe score of 1 compared to patients in other McCabe categories.

Table 2 describes the relative burden on female patients, patients aged less than 5 y, and patients aged 65 y and above for each HAI and overall (including and excluding HAI neonatal sepsis).

**Table 2:** Percentage of burden of healthcare-associated infections (% DALYs) in female patients, children (<15 y old), and the elderly (≥65 y old), EU/EEA, 2011–2012 (time discounting was not applied).

Healthcare-Associated Infections	Female Patients (% DALYs)	<15 y old (% DALYs)	≥65 y old (% DALYs)
HA Pneumonia	36.5	22.3	23.7
HA Primary Bloodstream Infection	44.3	41.2	11.6
HA Urinary Tract Infection	59.4	13.1	29.6
Surgical Site Infection	45.1	6.3	48.7
HA *C*. *difficile* Infection	53.3	17.8	31.9
**Overall, without HA Neonatal Sepsis**	**30.5**	**24.5**	**24.1**
HA Neonatal Sepsis	61.4	N/A	N/A
**Overall**	**40.8**	**27.2**	**23.3**

Abbreviations: N/A, not applicable.

**Fig 3:**
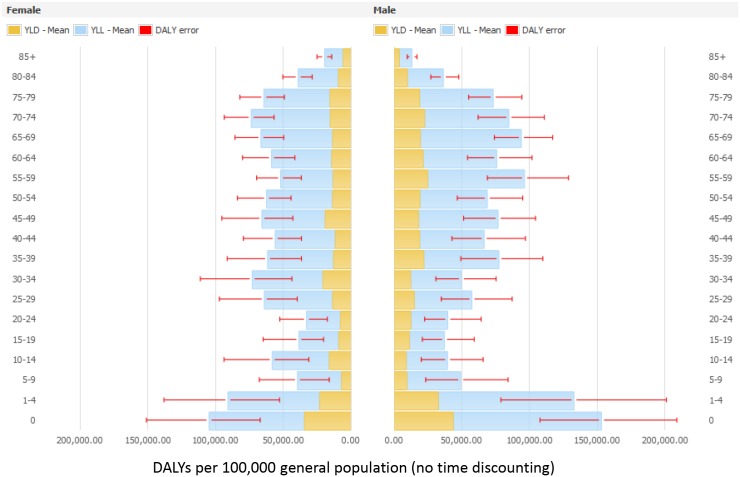
Estimated annual burden of six healthcare-associated infections in DALYs per 100,000 general population (median and 95% uncertainty interval) by gender and age group, split between YLLs and YLDs, EU/EEA, 2011–2012 (time discounting was not applied).

**Fig 4:**
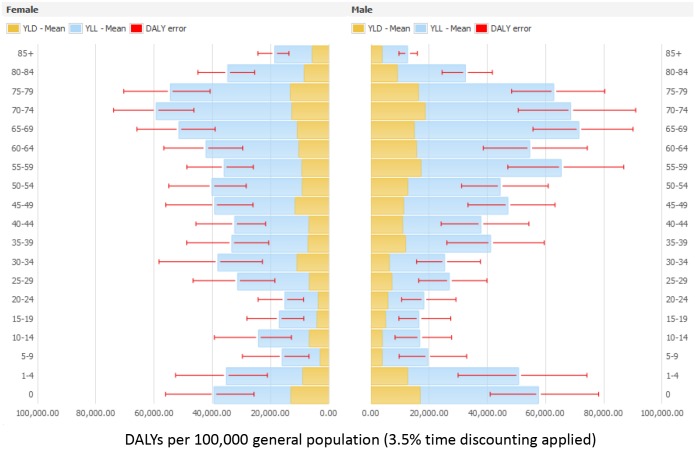
Estimated annual burden of six healthcare-associated infections in DALYs per 100,000 general population (median and 95% uncertainty interval) by gender and age group, split between YLLs and YLDs, EU/EEA, 2011–2012 (3.5% annual time discounting applied).

Figs 3 and 4 present the distribution of the burden of HAIs in DALYS per 100,000 total population by gender and by age group, without and with time discounting, respectively.

S1 Output provides detailed results for each HAI, as well as sensitivity analysis exploring the effect of lower values of ranges for each disease model.

## Discussion

**Fig 5:**
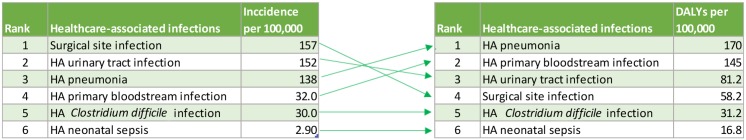
Ranking of six healthcare-associated infections according to their median incidence per 100,000 population and median DALYs per 100,000 population, EU/EEA, 2011–2012 (time discounting was not applied).

To our knowledge, this study represents the first attempt to estimate the burden of HAIs expressed in DALYs. Estimation of the burden of disease expressed in DALYs is a comprehensive and evidence-based approach to evaluate the burden of a disease that can be used to inform policy making in public health. The DALY is a composite measure that accounts not only for the number of cases but also for the associated mortality and short-term and long-term disabilities that result from a disease. DALYs provide a more comprehensive view of the burden of a disease, and the ranking of diseases according to DALYs is often different from the ranking based on incidence (Fig 5).

Despite the fact that the population at risk for HAIs was limited to hospitalised patients, our estimated total burden of HAIs in the EU/EEA of 501 DALYs per 100,000 general population was significantly higher than that of other communicable diseases as estimated by the BCoDE 2009–2013 study [27]. In comparison, the total burden of all other communicable diseases included in the BCoDE 2009–2013 study was 260 DALYs per 100,000 general population, including influenza (71.2 DALYs per 100,000 general population) and tuberculosis (53.5 DALYs per 100,000 general population).

The syndromic approach that we selected for estimating the burden of HAIs may partly explain this observation, and one should be cautious when making comparisons between infection syndromes and infections caused by specific microorganisms. However, this comparison is indicative of the relative burden of HAIs on population health and on the use of healthcare resources. Although HAIs are caused by various microorganisms and are associated with a number of risks and causation pathways, the specific syndromes are well defined, and a substantial proportion of HAI cases are preventable by common infection prevention and control measures.

HAP, including ventilator-associated pneumonia, and HA primary BSI were the HAIs with the highest health burden measured in DALYs, representing 60% of the total burden of HAIs under study. This is the result of a large number of cases combined with the severity of these HAIs. HA UTIs and HA SSIs represented almost 30% of the total burden of HAIs under study. The fact that more than half of the cases of these four types of HAI are considered preventable [1] and that the four cumulatively represent 90% of the burden of HAIs under study is an indication that lowering the burden of HAIs in the EU/EEA should be an achievable goal.

HA neonatal sepsis accounted for 1,592 DALYs per 100,000 newborns (852–2,580), 12 times the DALYs of the congenital infections (congenital toxoplasmosis, congenital chlamydia infections, congenital gonorrhoea, perinatal listeriosis, congenital rubella, and congenital syphilis) included in the BCoDE 2009–2013 study.

YLLs represented almost 75% of the total DALYs, and over 60% of the DALYs were due to the acute phase of the HAIs. This is due to the high in-hospital attributable mortality of HAIs that occurs mostly in the acute phase, while long-term consequences of HAIs are comparatively less significant and less well defined. The latter may also be due to the relative lack of available evidence.

Our estimates of the burden of HAIs in the EU/EEA must be placed in a broader perspective. The 2013 Global Burden of Disease (GBD 2013) estimated DALYs for a number of syndromes other than HAIs [28]. By downloading GBD 2013 country-specific estimates from the Global Health Data Exchange (GHDx) website and adding the number of DALYs in 2013, we were able to estimate the EU/EEA burden of cardiovascular diseases (5,097 DALYs per 100,000 general population), lower respiratory tract infections (LRTIs) (392 DALYs per 100,000), neonatal sepsis (11.4 DALYs per 100,000), and diarrhoea (37 DALYs per 100,000). Our estimate of the burden of HAP (169 DALYs per 100,000) was more than one third of the GBD 2013 burden of all LRTIs. Our estimate of the burden of HA neonatal sepsis was 1.5 times higher than that of neonatal sepsis reported by GBD 2013. However, GBD 2013 was a prevalence-based study, and its results did not account for the projected future burden of disease. When discounting our estimates, thus reducing the burden of long-term sequelae, we found that HA neonatal sepsis from our study was almost half of that of neonatal sepsis in GBD 2013.

The Ontario Burden of Infectious Disease Study (ONBOIDS) used a methodology comparable to that of our study and estimated the burden of several syndromes including pneumonia, septicaemia, and UTIs, but it did not limit itself to HA cases [29]. The resulting ranking of infections according to their burden was similar to our study, with pneumonia and septicaemia ranking first in terms of number of health-adjusted life years (HALYs), followed by UTIs. Moreover, as in our study, YLLs accounted for the largest part of the burden of disease, and the number of HALYs for *C*. *difficile* was surprisingly similar (27.2 HALYs per 100,000 general population in ONBOIDS versus 31.2 DALYs per 100,000 in our study).

Among the studied types of HAI, HA neonatal sepsis and HA primary BSI had the highest number of DALYs per case (12.1 and 8.0 DALYs per case, respectively), reflecting the severity of these infections for each affected patient. By comparison, in BCoDE 2009–2013, HIV/AIDS had 6.0 DALYs per case, invasive meningococcal disease had 5.6 DALYs per case, and tuberculosis had 3.6 DALYs per case.

In general, the burden of HAIs was higher in men, except for HA neonatal sepsis, HA UTI, and HA CDI, for which a higher proportion of the burden affected women. The fact that the incidence of HA CDI was higher in female patients is consistent with another study [30] and may be related to the higher incidence of HA CDI in elderly inpatients, a group in which female patients predominate. HAP, HA UTI, HA CDI, and, in particular, SSI had a higher burden on hospitalized patients aged 65 y and above, whereas HA primary BSI had a higher burden in paediatric patients aged less than 5 y. When including HA neonatal sepsis, almost half of the total burden of HAIs occurred in these more vulnerable population groups.

The present study was limited to HAIs in acute care settings. However, other studies indicate that, when long-term care facilities are included, the total number of HAIs each year approximately doubles [31]. Therefore, our results likely represent an underestimate of the total burden of HAIs on healthcare systems in the EU/EEA.

One strength of this study is the availability of data from the ECDC PPS to estimate the number of cases of HAIs in the EU/EEA. These data represent the most comprehensive assessment to date of the epidemiology of HAIs in the EU/EEA. An additional strength is the use of systematic literature reviews to determine attributable mortality, attributable length of hospital stay, and attributable short-term and long-term complications of HAIs. Lastly, the use of the McCabe score allowed adjustment of life expectancy, as a significant number of hospitalised patients have decreased life expectancy compared to the general population.

A number of limitations need to be taken into account when interpreting the results of this study. The outcome trees were developed based on systematic literature reviews and expert group consultations. The quality of evidence used to calculate the transitional probabilities varied as displayed in single study quality appraisals. For HA neonatal sepsis, we demonstrated by applying the GRADE (Grading of Recommendations, Assessment, Development and Evaluation) methodology that confidence in transitional probability estimates was also heterogeneous [16]. Variability in the estimates of HAI outcomes, and especially the attributable fraction of death associated with HAIs, was reflected in the range of the model parameters. Moreover, all outcome trees, except the one developed for SSI, were not adjusted for age-specific risks, assuming common transition probabilities for all subgroups.

Outcome trees were built on available published evidence, and the resulting disease progression pathway may not always fully reflect the definition of a case of HAI. However, while acknowledging that this might be a source of imprecision, the outcome trees were the best available approximation.

In the case of HA UTI, the outcome tree was based on studies of catheter-associated UTI in critically ill patients [32,33]. The diagnosis of HA UTI in these studies relied on bacteriuria. However, according to the surveillance definition for HA UTI used in the ECDC PPS [11], only symptomatic bacteriuria is considered a UTI. We estimated the transitional probability from symptomatic UTI to bacteraemia/urosepsis assuming that bacteraemia/urosepsis is more common in patients with symptomatic UTI and using data on the probability of development of symptomatic UTI in patients with catheter-associated bacteriuria [34]. This led to a four times higher burden compared to the use of transitional probabilities from bacteriuria to bacteraemia/sepsis and illustrates the challenges of devising transitional probabilities for the estimation of disease burden. A minor change of the interpretation of the source literature resulted in a significant change of the result. The case fatality proportion estimated for the HA UTI model of 0.5% to 4% indicates a 10-fold range of probability. This is reflected in the large UIs around the results for the burden of HA UTI, as shown in Table 1.

An additional limitation is the uncertainty of using the Rhame and Sudderth formula for estimation of the incidence of HAI from prevalence data. The Rhame and Sudderth method was designed for and has been applied specifically to HAI surveillance [35–37]. Although both under- and overestimation have been described with the use of this method, it is the most commonly used formula for this purpose, and its use therefore allows for comparisons with the results of similar studies.

Furthermore, we used Eurostat data for discharges of inpatients to calculate the total number of cases of HAI for each age group. Eurostat data are not fully comparable across EU/EEA Member States because of differences of healthcare provision and of inclusion of various types of healthcare facilities. However, for the majority of the countries, psychiatric, rehabilitation, and long-term care facilities are not included, and the number of hospital discharges mainly represent acute care hospitals. Thus, the inpatient population as defined in the Eurostat hospital discharges database is similar to the population of acute care hospitals studied in the ECDC PPS.

We only studied six selected types of HAI. These were chosen because of their severity, perceived burden, and availability of data. Other less frequent types of HAI—such as HA central nervous system infections or HA head-and-neck infections, which represented 22% of HAIs in the ECDC PPS—were not included. This may be a factor leading to an underestimation of the total burden of HAIs in the EU/EEA.

We did not address the burden of HAIs specifically associated with antimicrobial resistance, although multidrug-resistant microorganisms are often responsible for HAIs. The fraction of the burden of HAIs attributable to antimicrobial resistance is currently unknown but is expected to vary between EU/EEA Member States because of the large intercountry differences in antimicrobial resistance percentages [38]. Higher antimicrobial resistance percentages likely lead to increased morbidity and mortality due to inappropriate and ineffective treatment. In addition, the current increasing trends in antimicrobial resistance in bacteria responsible for HAI such as *Klebsiella pneumoniae* or *Acinetobacter* spp. combined with the lack of new antibiotics active against these bacteria likely contribute to increasing an already high burden of HAIs in the EU/EEA.

The present study highlights the substantial burden of HAIs in the EU/EEA, compared to other communicable diseases under surveillance in the EU, and the need for intensified efforts to prevent and control these infections, ultimately making European hospitals safer places.

## Supporting Information

S1 Input Annual number of age-group- and sex-specific cases per HAI and McCabe score. (PDF)

S1 Models Disease outcome trees. (PDF)

S1 Output HAI detailed results and sensitivity analysis. (XLSX)

## Abbreviations

BCoDE: Burden of Communicable Diseases in Europe
CABG: coronary artery bypass graft
DALY: disability-adjusted life year
ECDC: European Centre for Disease Prevention and Control
EU/EEA: European Union and European Economic Area
GBD: Global Burden of Disease
GHDx: Global Health Data Exchange
GRADE: Grading of Recommendations, Assessment, Development and Evaluation
HA: healthcare-associated
HA CDI: healthcare-associated *Clostridium difficile* infection
HAI: healthcare-associated infection
HALY: health-adjusted life year
HAP: healthcare-associated pneumonia
HA primary BSI: healthcare-associated primary bloodstream infection
HA UTI: healthcare-associated urinary tract infection
LRTI: lower respiratory tract infection
ONBOIDS: Ontario Burden of Infectious Disease Study
PERT: Project Evaluation and Review Techniques
PPS: point prevalence survey
SSI: surgical site infection
UI: uncertainty interval
YLDs: years lived with disability
YLLs: years of life lost due to premature mortality

## References

[1] Umscheid CA, Mitchell MD, Doshi JA, Agarwal R, Williams K, Brennan PJ. Estimating the proportion of healthcare-associated infections that are reasonably preventable and the related mortality and costs. Infect Control Hosp Epidemiol. 2011; 32: 101–114. 10.1086/657912 21460463

[2] World Health Organization (WHO). Report on the burden of endemic health care-associated infection worldwide. Geneva: WHO; 2011 http://apps.who.int/iris/bitstream/10665/80135/1/9789241501507_eng.pdf.

[3] Magill SS, Edwards JR, Bamberg W, Beldavs ZG, Dumyati G, Kainer MA, et al Multistate point-prevalence survey of health care-associated infections. N Engl J Med. 2014; 370: 1198–1208. 10.1056/NEJMoa1306801 24670166 PMC4648343

[4] Zarb P, Coignard B, Griskeviciene J, Muller A, Vankerckhoven V, Weist K, et al The European Centre for Disease Prevention and Control (ECDC) pilot point prevalence survey of healthcare-associated infections and antimicrobial use. Euro Surveill. 2012; 17(46). http://www.eurosurveillance.org/ViewArticle.aspx?ArticleId=20316. 23171822 10.2807/ese.17.46.20316-en

[5] European Centre for Disease prevention and Control. Annual epidemiological report on communicable diseases in Europe 2008 Stockholm: ECDC; 2009 http://ecdc.europa.eu/en/publications/Publications/0812_SUR_Annual_Epidemiological_Report_2008.pdf.

[6] European Centre for Disease prevention and Control. Point prevalence survey of healthcare-associated infections and antimicrobial use in European acute care hospitals. Stockholm: ECDC; 2013 http://ecdc.europa.eu/en/publications/Publications/healthcare-associated-infections-antimicrobial-use-PPS.pdf.

[7] Muray CJL, Salomon JA, Marthers CD, Lopez AD. Summary measures of population health. Geneva: WHO; 2002 http://apps.who.int/iris/bitstream/10665/42439/1/9241545518.pdf.

[8] Sherman ER, Heydon KH, St John KH, Teszner E, Rettig SL, Alexander SK, et al Administrative data fail to accurately identify cases of healthcare-associated infection. Infect Control Hosp Epidemiol. 2006; 27: 332–337. 10.1086/502684 16622808

[9] Mangen MJ, Plass D, Havelaar AH, Gibbons CL, Cassini A, Muhlberger N, et al The pathogen- and incidence-based DALY approach: an appropriate [corrected] methodology for estimating the burden of infectious diseases. PLoS ONE. 2013; 8: e79740 10.1371/journal.pone.0079740 24278167 PMC3835936

[10] Kretzschmar M, Mangen MJ, Pinheiro P, Jahn B, Fevre EM, Longhi S, et al New methodology for estimating the burden of infectious diseases in Europe. PLoS Med. 2012; 9: e1001205 10.1371/journal.pmed.1001205 22529750 PMC3328443

[11] European Centre for Disease prevention and Control. Point prevalence survey of healthcare-associated infections and antimicrobial use in European acute care hospitals—protocol version 4.3. Stockholm: ECDC; 2012 http://ecdc.europa.eu/en/publications/Publications/0512-TED-PPS-HAI-antimicrobial-use-protocol.pdf.

[12] Murray CJ, Ezzati M, Flaxman AD, Lim S, Lozano R, Michaud C, et al GBD 2010: design, definitions, and metrics. Lancet. 2012; 380: 2063–2066. 10.1016/S0140-6736(12)61899-6 23245602

[13] Haagsma JA, Polinder S, Cassini A, Colzani E, Havelaar AH. Review of disability weight studies: comparison of methodological choices and values. Popul Health Metr. 2014; 12: 20 10.1186/s12963-014-0020-2 26019690 PMC4445691

[14] Salomon JA, Haagsma JA, Davis A, de Noordhout CM, Polinder S, Havelaar AH, et al Disability weights for the Global Burden of Disease 2013 study. Lancet Glob Health. 2015; 3: e712–723. 10.1016/S2214-109X(15)00069-8 26475018

[15] Haagsma JA, Maertens de Noordhout C, Polinder S, Vos T, Havelaar AH, Cassini A, et al Assessing disability weights based on the responses of 30,660 people from four European countries. Popul Health Metr. 2015; 13: 1–15. 10.1186/s12963-015-0042-4 26778920 PMC4715333

[16] Haller S, Deindl P, Cassini A, Suetens C, Zingg W, Abu Sin M, et al Neurological sequelae of healthcare-associated sepsis in very-low-birthweight infants: Umbrella review and evidence-based outcome tree. Euro Surveill. 2016 2 25; 21(8). 10.2807/1560-7917.ES.2016.21.8.30143 http://www.eurosurveillance.org/ViewArticle.aspx?ArticleId=21389. 26940884

[17] Robert Koch Institute. Burden of healthcare associated infection (BHAI)—evidence-based and comorbidity-adjusted outcome trees for estimation of burden of disease. Berlin: RKI; 2016 http://www.rki.de/DE/Content/Institut/OrgEinheiten/Abt3/FG37/Research_Report_BHAI.pdf?__blob=publicationFile.

[18] European Centre for Disease prevention and Control. ECDC BCoDE toolkit [software application]. Stockholm: European Centre for Disease Prevention and Control; 2015 version 1.4: http://ecdc.europa.eu/en/healthtopics/burden_of_communicable_diseases/Pages/Tool.aspx.

[19] McCabe WR. Gram-Negative Bacteremia. Arch Intern Med. 1962; 110: 847 10.1001/archinte.1962.03620240029006

[20] Astagneau P, Rioux C, Golliot F, Brucker G, Group INS. Morbidity and mortality associated with surgical site infections: results from the 1997–1999 INCISO surveillance. J Hosp Infect. 2001; 48: 267–274. 10.1053/jhin.2001.1003 11461127

[21] Eurostat. Hospital beds by type of care. 2013. http://ec.europa.eu/eurostat/web/health/health-care/data/database.

[22] Eurostat. Hospital discharges by diagnosis, in-patients, per 100 000 inhabitants. 2011. http://ec.europa.eu/eurostat/web/health/health-care/data/database.

[23] Rhame FS, Sudderth WD. Incidence and prevalence as used in the analysis of the occurrence of nosocomial infections. Am J Epidemiol. 1981; 113: 1–11. 7457475 10.1093/oxfordjournals.aje.a113058

[24] Gibbons CL, Mangen MJ, Plass D, Havelaar AH, Brooke RJ, Kramarz P, et al Measuring underreporting and under-ascertainment in infectious disease datasets: a comparison of methods. BMC Public Health. 2014; 14: 147 10.1186/1471-2458-14-147 24517715 PMC4015559

[25] Vose D Risk analysis—a quantitative guide. Chichester: John Wiley & Sons, LTD; 2001.

[26] National Institute for Health and Care Excellence (NICE). Guide to the methods of technology appraisal. 2013. https://www.nice.org.uk/process/pmg9/resources/guide-to-the-methods-of-technology-appraisal-2013-pdf-2007975843781 27905712

[27] Colzani E. Results from the 2015 Burden of Communicable Diseases in Europe (BCoDE) study. European Public Health Conference; Milan, 2015.

[28] Global Burden of Disease Study. Global, regional, and national incidence, prevalence, and years lived with disability for 301 acute and chronic diseases and injuries in 188 countries, 1990–2013: a systematic analysis for the Global Burden of Disease Study 2013. Lancet. 2015; 386: 743–800. 10.1016/S0140-6736(15)60692-4 26063472 PMC4561509

[29] Kwong JC, Ratnasingham S, Campitelli MA, Daneman N, Deeks SL, Manuel DG, et al The impact of infection on population health: results of the Ontario burden of infectious diseases study. PLoS ONE. 2012; 7: e44103 10.1371/journal.pone.0044103 22962601 PMC3433488

[30] Thomas C, Stevenson M, Williamson DJ, Riley TV. Clostridium difficile-associated diarrhea: epidemiological data from Western Australia associated with a modified antibiotic policy. Clin Infect Dis. 2002; 35: 1457–1462. 10.1086/342691 12471563

[31] Suetens C. Healthcare-associated infections in European long-term care facilities: how big is the challenge? Euro Surveill. 2012; 17(35). http://www.eurosurveillance.org/ViewArticle.aspx?ArticleId=20259. 22958606

[32] Clec'h C, Schwebel C, Francais A, Toledano D, Fosse JP, Garrouste-Orgeas M, et al Does catheter-associated urinary tract infection increase mortality in critically ill patients? Infect Control Hosp Epidemiol. 2007; 28: 1367–1373. 10.1086/523279 17994517

[33] Laupland KB, Zygun DA, Davies HD, Church DL, Louie TJ, Doig CJ. Incidence and risk factors for acquiring nosocomial urinary tract infection in the critically ill. J Crit Care. 2002; 17: 50–57. 12040549 10.1053/jcrc.2002.33029

[34] Saint S. Clinical and economic consequences of nosocomial catheter-related bacteriuria. Am J Infect Control. 2000; 28: 68–75. 10.1016/S0196-6553(00)90015-4 10679141

[35] Gastmeier P, Brauer H, Sohr D, Geffers C, Forster DH, Daschner F, et al Converting incidence and prevalence data of nosocomial infections: results from eight hospitals. Infect Control Hosp Epidemiol. 2001; 22: 31–34. 10.1086/501821 11198019

[36] Berthelot P, Garnier M, Fascia P, Guyomarch S, Jospe R, Lucht F, et al Conversion of prevalence survey data on nosocomial infections to incidence estimates: a simplified tool for surveillance? Infect Control Hosp Epidemiol. 2007; 28: 633–636. 10.1086/513536 17464932

[37] Rossello-Urgell J, Rodriguez-Pla A. Behavior of cross-sectional surveys in the hospital setting: a simulation model. Infect Control Hosp Epidemiol. 2005; 26: 362–368. 10.1086/502553 15865272

[38] European Centre for Disease prevention and Control. Antimicrobial resistance surveillance in Europe 2014 Annual Report of the European Antimicrobial Resistance Surveillance Network (EARS-Net). Stockholm: ECDC; 2015 http://ecdc.europa.eu/en/publications/Publications/antimicrobial-resistance-europe-2014.pdf.

