# Supplementary material for: Burden of Six Healthcare-Associated Infections on European Population Health: Estimating Incidence-Based Disability-Adjusted Life Years through a Population Prevalence-Based Modelling Study
Source: PLoS Med. 2016 Oct 18;13(10):e1002150. doi: 10.1371/journal.pmed.1002150 (PMC5068791; doi:10.1371/journal.pmed.1002150)
Supplement: S1 Input — (PDF) [file pmed.1002150.s001.pdf]

Burden of Communicable Diseases in Europe Project

Office of the Chief Scientist Unit

Supplementary Information 2
Healthcare-associated infections
Input tables – annual number of age-group and sex specific cases per HAI infection and McCabe score

Produced by Alessandro Cassini, Diamantis Plachouras and Carl Suetens (ECDC)

Healthcare-associated *Clostridium difficile* infection (HA CDI)

2011-2012 HA CDI annual number of cases - McCabe 1						
Age group	Females			Males		
	2.5 %	Median	97.5 %	2.5 %	Median	97.5 %
0	0.00	**0.00**	1,067.75	76.15	**628.75**	2,270.22
01 - 04	23.83	**941.26**	5,236.97	0.00	**0.00**	4,059.18
05 - 09	0.00	**0.00**	5,040.99	163.38	**1,348.68**	4,861.73
10 - 14	0.00	**0.00**	4,240.13	0.00	**0.00**	3,611.40
15 - 19	0.00	**0.00**	2,178.54	0.00	**0.00**	2,664.31
20 - 24	0.00	**0.00**	3,408.81	0.00	**0.00**	2,675.70
25 - 29	240.02	**1,981.73**	7,154.30	124.77	**1,029.99**	3,714.57
30 - 34	269.30	**2,223.49**	8,027.51	0.00	**0.00**	2,050.76
35 - 39	200.87	**1,658.52**	5,987.08	18.01	**711.17**	3,958.32
40 - 44	351.88	**1,705.88**	4,980.50	0.00	**0.00**	2,203.41
45 - 49	174.34	**1,439.41**	5,195.51	854.84	**2,631.78**	6,135.50
50 - 54	115.65	**954.84**	3,446.84	583.27	**2,140.20**	5,475.47
55 - 59	307.81	**1,492.37**	4,358.31	1,316.51	**3,273.44**	6,739.47
60 - 64	533.93	**1,959.23**	5,013.05	699.30	**2,153.26**	5,022.06
65 - 69	1,572.76	**3,641.72**	7,170.41	1,157.71	**2,878.69**	5,927.32
70 - 74	2,296.79	**4,599.32**	8,223.76	2,236.61	**4,662.46**	8,568.49
75 - 79	3,966.75	**6,806.51**	10,890.02	4,524.72	**7,912.10**	12,837.77
80 - 84	5,476.87	**8,458.18**	12,474.88	3,076.26	**5,492.95**	9,050.09
85+	5,792.15	**8,711.69**	12,580.21	2,396.24	**4,497.24**	7,680.81
**Total**	21,322.95	**46,574.15**	116,675.59	17,227.76	**39,360.70**	99,506.58

2011-2012 HA CDI annual number of cases - McCabe 2						
Age group	Females			Males		
	2.5 %	Median	97.5 %	2.5 %	Median	97.5 %
0	0.00	**0.00**	605.54	0.00	**0.00**	497.31
01 - 04	0.00	**0.00**	1,915.17	13.82	**546.11**	2,985.87
05 - 09	0.00	**0.00**	3,815.25	0.00	**0.00**	1,874.88
10 - 14	0.00	**0.00**	1,949.33	0.00	**0.00**	1,121.16
15 - 19	0.00	**0.00**	1,565.78	0.00	**0.00**	1,473.68
20 - 24	0.00	**0.00**	2,243.81	0.00	**0.00**	780.28
25 - 29	0.00	**0.00**	1,616.11	0.00	**0.00**	577.90
30 - 34	145.96	**1,201.55**	4,249.18	0.00	**0.00**	465.15
35 - 39	0.00	**0.00**	1,891.66	4.60	**181.72**	1,002.54
40 - 44	9.06	**357.92**	1,977.90	0.00	**0.00**	947.07
45 - 49	55.22	**455.58**	1,635.85	5.60	**221.13**	1,226.90
50 - 54	78.65	**649.03**	2,334.41	0.00	**0.00**	951.95
55 - 59	72.62	**599.36**	2,158.82	388.21	**1,194.48**	2,780.09
60 - 64	179.28	**868.85**	2,532.82	346.41	**1,066.18**	2,483.68
65 - 69	727.19	**1,979.52**	4,297.55	581.97	**1,446.39**	2,974.81
70 - 74	2,454.14	**4,377.74**	7,200.21	251.15	**921.38**	2,355.97
75 - 79	1,967.81	**3,595.18**	6,019.67	346.98	**1,068.14**	2,489.51
80 - 84	616.32	**1,532.09**	3,152.64	1,009.83	**2,104.12**	3,863.22
85+	3,208.13	**4,952.59**	7,300.33	1,148.32	**2,154.32**	3,676.78
**Total**	9,514.39	**20,569.40**	58,462.04	4,096.89	**10,903.96**	34,528.77

2011-2012 HA CDI annual number of cases - McCabe 3						
Age group	Females			Males		
	2.5 %	Median	97.5 %	2.5 %	Median	97.5 %
0	0.00	**0.00**	287.92	0.00	**0.00**	544.01
01 - 04	0.00	**0.00**	202.02	0.00	**0.00**	1,165.26
05 - 09	5.47	**216.58**	912.24	0.00	**0.00**	1,326.82
10 - 14	0.00	**0.00**	452.46	0.00	**0.00**	1,495.09
15 - 19	0.00	**0.00**	1,336.73	0.00	**0.00**	304.50
20 - 24	0.00	**0.00**	1,776.61	0.00	**0.00**	946.11
25 - 29	0.00	**0.00**	8,674.31	0.00	**0.00**	264.79
30 - 34	0.00	**0.00**	2,275.88	0.00	**0.00**	685.43
35 - 39	0.00	**0.00**	1,205.56	6.72	**265.52**	1,428.40
40 - 44	5.66	**223.51**	1,218.52	3.05	**120.60**	657.08
45 - 49	8.13	**320.97**	1,758.58	0.00	**0.00**	676.41
50 - 54	0.00	**0.00**	711.37	5.16	**203.65**	1,126.66
55 - 59	0.00	**0.00**	691.70	5.38	**212.55**	1,178.64
60 - 64	0.00	**0.00**	980.43	109.37	**529.80**	1,541.39
65 - 69	144.01	**697.21**	2,023.99	158.12	**579.52**	1,477.10
70 - 74	157.41	**576.71**	1,468.23	4.58	**181.08**	1,005.78
75 - 79	323.27	**993.99**	2,309.05	184.01	**674.62**	1,721.20
80 - 84	193.69	**710.03**	1,810.92	88.63	**429.39**	1,250.24
85+	418.26	**1,039.10**	2,135.11	222.67	**684.79**	1,591.68
**Total**	1,255.91	**4,778.11**	32,231.64	787.69	**3,881.51**	20,386.58

Healthcare-associated primary bloodstream infection (HA primary BSI)

2011-2012 HA primary BSI annual number of cases - McCabe 1						
Age group	Females			Males		
	2.5 %	Median	97.5 %	2.5 %	Median	97.5 %
0	1,040.03	**2,082.48**	3,722.93	2,766.19	**4,465.97**	6,820.12
01 - 04	665.95	**2,442.71**	6,242.27	1,849.16	**4,595.69**	9,451.56
05 - 09	171.11	**1,412.31**	5,087.50	172.55	**1,424.37**	5,134.57
10 - 14	155.47	**1,283.32**	4,624.23	18.66	**737.05**	4,097.83
15 - 19	149.49	**1,234.11**	4,451.33	0.00	**0.00**	2,407.73
20 - 24	0.00	**0.00**	2,217.05	0.00	**0.00**	2,120.85
25 - 29	362.90	**1,759.43**	5,138.33	337.74	**1,637.01**	4,775.45
30 - 34	0.00	**0.00**	2,149.76	327.69	**1,588.41**	4,634.87
35 - 39	565.84	**2,076.23**	5,311.69	808.46	**2,488.58**	5,798.97
40 - 44	824.47	**2,538.21**	5,916.76	815.21	**2,509.55**	5,849.09
45 - 49	291.86	**1,414.94**	4,131.45	1,312.19	**3,262.18**	6,713.90
50 - 54	1,805.34	**3,946.14**	7,483.11	729.15	**2,244.96**	5,234.64
55 - 59	651.23	**2,005.09**	4,675.51	3,142.56	**5,745.06**	9,630.09
60 - 64	1,820.50	**3,794.65**	6,972.30	1,328.30	**3,075.76**	6,056.41
65 - 69	948.13	**2,357.53**	4,854.07	2,870.47	**5,248.00**	8,797.99
70 - 74	2,341.63	**4,396.02**	7,511.86	621.35	**1,913.27**	4,462.50
75 - 79	1,710.14	**3,424.70**	6,124.01	1,238.83	**2,868.60**	5,648.58
80 - 84	1,179.49	**2,578.72**	4,892.30	532.67	**1,640.10**	3,824.67
85+	63.05	**520.64**	1,880.06	464.76	**1,430.90**	3,336.27
**Total**	14,746.64	**39,267.26**	93,386.52	19,335.93	**46,875.46**	104,796.07

2011-2012 HA primary BSI annual number of cases - McCabe 2						
Age group	Females			Males		
	2.5 %	Median	97.5 %	2.5 %	Median	97.5 %
0	26.11	**214.91**	759.48	215.14	**656.25**	1,490.98
01 - 04	234.67	**1,129.88**	3,206.06	0.00	**0.00**	1,444.70
05 - 09	91.86	**754.53**	2,628.50	0.00	**0.00**	1,695.39
10 - 14	0.00	**0.00**	1,676.97	9.72	**384.18**	2,062.01
15 - 19	12.25	**483.98**	2,621.67	0.00	**0.00**	1,154.42
20 - 24	12.30	**485.83**	2,638.38	0.00	**0.00**	881.29
25 - 29	124.28	**1,022.72**	3,609.22	43.12	**354.84**	1,252.51
30 - 34	139.64	**1,149.59**	4,066.81	4.18	**165.11**	906.72
35 - 39	11.74	**463.76**	2,551.84	0.00	**0.00**	721.49
40 - 44	391.52	**1,432.11**	3,626.49	6.84	**270.16**	1,494.77
45 - 49	77.70	**641.06**	2,302.45	1,240.21	**2,576.64**	4,703.50
50 - 54	207.49	**1,005.01**	2,923.47	1,116.12	**2,322.40**	4,252.42
55 - 59	1,869.27	**3,501.04**	5,957.22	381.11	**1,172.67**	2,729.61
60 - 64	450.89	**1,387.25**	3,228.25	1,457.67	**2,734.03**	4,664.15
65 - 69	1,044.46	**2,281.01**	4,317.70	216.48	**794.16**	2,030.42
70 - 74	1,032.55	**2,255.66**	4,272.27	1,240.90	**2,399.16**	4,183.18
75 - 79	636.34	**1,581.66**	3,253.69	1,727.85	**3,019.61**	4,894.54
80 - 84	124.47	**603.41**	1,761.70	843.01	**1,756.55**	3,225.18
85+	4.35	**171.97**	957.63	458.93	**1,140.70**	2,346.63
**Total**	6,491.91	**20,565.38**	56,359.82	8,961.28	**19,746.46**	46,133.89

2011-2012 HA primary BSI annual number of cases - McCabe 3						
Age group	Females			Males		
	2.5 %	Median	97.5 %	2.5 %	Median	97.5 %
0	0.00	**0.00**	311.50	0.00	**0.00**	371.06
01 - 04	6.60	**260.74**	1,261.25	87.12	**701.92**	2,153.37
05 - 09	0.00	**0.00**	992.41	8.35	**329.99**	1,498.35
10 - 14	0.00	**0.00**	797.18	0.00	**0.00**	835.47
15 - 19	0.00	**0.00**	1,203.19	0.00	**0.00**	850.29
20 - 24	0.00	**0.00**	1,506.40	0.00	**0.00**	545.67
25 - 29	0.00	**0.00**	2,354.23	60.74	**290.05**	795.93
30 - 34	0.00	**0.00**	1,490.34	3.32	**131.18**	702.71
35 - 39	78.16	**641.41**	2,221.37	0.00	**0.00**	550.63
40 - 44	6.31	**249.20**	1,360.68	245.41	**892.81**	2,221.79
45 - 49	0.00	**0.00**	1,019.35	111.02	**536.63**	1,547.83
50 - 54	67.93	**559.86**	1,996.04	289.66	**888.94**	2,054.03
55 - 59	504.47	**1,368.59**	2,945.98	192.56	**705.36**	1,794.79
60 - 64	135.32	**655.06**	1,901.02	102.54	**496.70**	1,445.39
65 - 69	442.24	**1,201.39**	2,594.85	335.28	**911.83**	1,974.93
70 - 74	542.54	**1,345.71**	2,755.00	98.49	**477.14**	1,389.18
75 - 79	111.98	**542.51**	1,579.45	399.17	**991.12**	2,033.93
80 - 84	4.14	**163.41**	907.97	3.33	**131.55**	730.84
85+	387.97	**963.86**	1,980.57	271.23	**737.88**	1,599.40
**Total**	2,287.64	**7,951.74**	31,178.80	2,208.19	**8,223.10**	25,095.57

Healthcare-associated pneumonia (HAP)

2011-2012 HAP annual number of cases - McCabe 1						
Age group	Females			Males		
	2.5 %	Median	97.5 %	2.5 %	Median	97.5 %
0	1,590.73	**3,185.14**	5,694.17	5,343.09	**8,173.74**	11,963.79
01 - 04	2,074.36	**5,647.59**	12,265.57	3,102.16	**7,709.73**	15,855.77
05 - 09	0.00	**0.00**	5,040.99	417.43	**2,023.02**	5,898.50
10 - 14	1,256.10	**4,605.88**	11,757.64	607.13	**2,942.10**	8,574.72
15 - 19	644.69	**2,364.80**	6,043.92	2,038.19	**5,063.11**	10,401.52
20 - 24	23.41	**924.72**	5,147.79	2,511.66	**5,810.34**	11,417.13
25 - 29	613.12	**2,972.60**	8,681.31	3,570.47	**6,694.93**	11,415.32
30 - 34	3,129.91	**7,782.20**	16,021.58	2,292.55	**5,008.36**	9,486.66
35 - 39	20.99	**829.26**	4,617.60	7,597.42	**12,800.98**	20,183.92
40 - 44	2,341.64	**5,117.65**	9,701.73	7,780.56	**12,552.40**	19,146.96
45 - 49	7,685.21	**12,954.71**	20,441.41	6,436.84	**10,527.11**	16,231.49
50 - 54	3,656.72	**6,683.89**	11,200.39	9,096.50	**13,911.31**	20,352.32
55 - 59	4,929.97	**8,456.74**	13,523.48	13,773.62	**19,172.98**	25,971.83
60 - 64	4,853.79	**8,326.72**	13,317.29	14,886.98	**20,240.63**	26,879.29
65 - 69	7,004.96	**10,925.15**	16,237.91	14,923.20	**20,150.83**	26,603.32
70 - 74	12,312.46	**17,142.93**	23,229.19	26,711.46	**34,035.98**	42,728.60
75 - 79	16,256.42	**21,620.68**	28,177.47	25,707.85	**33,131.91**	42,011.07
80 - 84	10,537.04	**14,548.06**	19,574.39	16,470.93	**21,605.60**	27,817.17
85+	14,258.34	**18,667.91**	24,001.29	12,560.57	**16,951.13**	22,362.35
**Total**	93,189.87	**152,756.63**	254,675.10	175,828.62	**258,506.19**	375,301.72

2011-2012 HAP annual number of cases - McCabe 2						
Age group	Females			Males		
	2.5 %	Median	97.5 %	2.5 %	Median	97.5 %
0	103.86	**500.54**	1,425.88	224.63	**684.74**	1,552.85
01 - 04	582.61	**2,116.85**	5,245.92	1,216.67	**3,276.66**	6,925.54
05 - 09	26.88	**1,061.75**	5,726.08	325.40	**1,563.07**	4,392.94
10 - 14	134.92	**1,103.96**	3,747.38	7.94	**313.70**	1,680.46
15 - 19	105.70	**868.64**	3,037.03	99.07	**814.77**	2,862.54
20 - 24	15.73	**621.40**	3,371.59	52.56	**432.08**	1,514.65
25 - 29	11.28	**445.63**	2,430.97	175.32	**637.61**	1,585.00
30 - 34	145.96	**1,201.55**	4,249.18	3.23	**127.64**	700.50
35 - 39	567.65	**2,072.29**	5,214.50	632.49	**1,453.74**	2,816.67
40 - 44	221.99	**1,073.77**	3,105.61	571.13	**1,549.40**	3,334.95
45 - 49	503.19	**1,366.73**	2,950.71	1,537.85	**2,874.69**	4,874.18
50 - 54	915.72	**2,271.60**	4,651.69	3,374.07	**5,430.05**	8,250.69
55 - 59	2,523.79	**4,495.18**	7,374.08	4,655.79	**6,927.96**	9,899.34
60 - 64	3,776.26	**6,081.92**	9,252.25	7,938.81	**10,661.84**	13,996.26
65 - 69	5,619.79	**8,577.92**	12,513.39	8,587.36	**11,364.51**	14,733.34
70 - 74	6,890.67	**9,922.87**	13,812.30	10,172.86	**13,359.94**	17,208.46
75 - 79	7,573.07	**10,528.74**	14,237.97	14,166.11	**17,731.16**	21,896.44
80 - 84	8,328.51	**11,162.37**	14,635.61	13,388.33	**16,832.93**	20,869.82
85+	12,237.34	**15,452.08**	19,239.03	6,308.82	**8,451.56**	11,074.51
**Total**	50,284.92	**80,925.80**	136,221.17	73,438.44	**104,488.04**	150,169.13

2011-2012 HAP annual number of cases - McCabe 3						
Age group	Females			Males		
	2.5 %	Median	97.5 %	2.5 %	Median	97.5 %
0	135.24	**405.78**	881.28	0.00	**0.00**	544.01
01 - 04	0.00	**0.00**	202.02	0.00	**0.00**	1,165.26
05 - 09	0.00	**0.00**	640.06	10.88	**430.09**	1,913.98
10 - 14	4.38	**173.42**	621.19	13.71	**542.57**	2,087.50
15 - 19	0.00	**0.00**	1,336.73	0.00	**0.00**	304.50
20 - 24	13.19	**521.27**	2,631.45	308.08	**1,092.54**	2,508.08
25 - 29	65.80	**2,600.65**	12,776.97	83.79	**300.24**	711.85
30 - 34	403.82	**1,927.77**	5,282.59	120.38	**576.33**	1,597.95
35 - 39	82.30	**675.00**	2,329.09	943.43	**2,124.17**	3,938.24
40 - 44	54.33	**447.01**	1,575.08	75.12	**361.80**	1,027.82
45 - 49	77.96	**641.95**	2,274.82	645.05	**1,480.62**	2,860.44
50 - 54	120.86	**583.99**	1,681.71	1,724.89	**3,054.78**	4,962.13
55 - 59	417.28	**1,131.85**	2,435.24	3,467.09	**5,313.64**	7,746.91
60 - 64	754.34	**1,868.88**	3,815.65	3,453.07	**5,121.40**	7,282.76
65 - 69	2,861.85	**4,648.08**	7,087.88	4,172.02	**5,795.16**	7,808.17
70 - 74	1,108.95	**2,018.48**	3,357.39	4,596.34	**6,518.72**	8,939.80
75 - 79	2,130.07	**3,578.37**	5,614.80	6,890.83	**9,107.32**	11,771.48
80 - 84	3,030.02	**4,615.22**	6,711.36	3,390.83	**4,866.42**	6,741.31
85+	3,137.03	**4,601.75**	6,500.49	2,786.91	**4,108.73**	5,820.16
**Total**	14,397.42	**30,439.44**	67,755.81	32,682.42	**50,794.55**	79,732.34

Healthcare-associated neonatal sepsis

2011-2012 Healthcare-associated neonatal sepsis annual number of cases - McCabe 1						
Age group	Females			Males		
	2.5 %	Median	97.5 %	2.5 %	Median	97.5 %
0	2155	**4314**	7708	1053	**2619**	5390
01 - 04						
05 - 09						
10 - 14						
15 - 19						
20 - 24						
25 - 29						
30 - 34						
35 - 39						
40 - 44						
45 - 49						
50 - 54						
55 - 59						
60 - 64						
65 - 69						
70 - 74						
75 - 79						
80 - 84						
85+						
**Total**	2,155.00	**4,314.00**	7,708.00	1,053.00	**2,619.00**	5,390.00

2011-2012 Healthcare-associated neonatal sepsis annual number of cases - McCabe 2						
Age group	Females			Males		
	2.5 %	Median	97.5 %	2.5 %	Median	97.5 %
0	0	**0**	4270	34	**1352**	6924
01 - 04						
05 - 09						
10 - 14						
15 - 19						
20 - 24						
25 - 29						
30 - 34						
35 - 39						
40 - 44						
45 - 49						
50 - 54						
55 - 59						
60 - 64						
65 - 69						
70 - 74						
75 - 79						
80 - 84						
85+						
**Total**	0.00	**0.00**	4,270.00	34.00	**1,352.00**	6,924.00

2011-2012 Healthcare-associated neonatal sepsis annual number of cases - McCabe 3						
Age group	Females			Males		
	2.5 %	Median	97.5 %	2.5 %	Median	97.5 %
0	39	**1537**	7368	250	**2028**	6570
01 - 04						
05 - 09						
10 - 14						
15 - 19						
20 - 24						
25 - 29						
30 - 34						
35 - 39						
40 - 44						
45 - 49						
50 - 54						
55 - 59						
60 - 64						
65 - 69						
70 - 74						
75 - 79						
80 - 84						
85+						
**Total**	39.00	**1,537.00**	7,368.00	250.00	**2,028.00**	6,570.00

Healthcare-associated surgical site infection (HA SSI)

2011-2012 HA SSI annual number of cases - McCabe 1						
Age group	Females			Males		
	2.5 %	Median	97.5 %	2.5 %	Median	97.5 %
0	406.94	**1,108.58**	2,411.22	226.02	**829.40**	2,122.44
01 - 04	368.47	**1,786.00**	5,210.14	1,807.16	**4,491.30**	9,236.78
05 - 09	166.75	**1,376.37**	4,958.01	1,126.36	**3,465.64**	8,066.07
10 - 14	1,016.15	**3,125.95**	7,271.63	5,010.79	**9,388.11**	15,984.13
15 - 19	1,325.41	**3,608.72**	7,838.64	4,898.44	**8,940.99**	14,945.51
20 - 24	4,922.63	**8,786.02**	14,464.83	5,181.55	**9,046.31**	14,638.52
25 - 29	6,538.37	**10,851.52**	16,924.73	4,096.50	**7,480.41**	12,513.42
30 - 34	8,743.81	**13,635.75**	20,263.48	8,877.74	**13,560.92**	19,804.82
35 - 39	6,572.68	**10,608.32**	16,192.62	10,719.83	**15,640.33**	22,014.52
40 - 44	14,236.18	**19,892.14**	27,021.66	12,900.36	**18,287.03**	25,138.75
45 - 49	13,607.37	**18,931.22**	25,624.99	21,490.12	**28,037.66**	35,920.78
50 - 54	19,794.26	**25,893.72**	33,254.39	17,183.37	**22,972.62**	30,064.18
55 - 59	19,446.61	**25,209.71**	32,123.69	27,796.47	**34,694.02**	42,759.05
60 - 64	21,048.93	**26,859.33**	33,757.09	33,583.03	**40,872.91**	49,250.70
65 - 69	19,235.97	**24,463.39**	30,657.02	29,822.97	**36,636.83**	44,519.03
70 - 74	25,300.76	**31,266.20**	38,197.59	23,905.13	**30,108.67**	37,412.15
75 - 79	21,620.09	**26,925.40**	33,124.91	25,609.49	**31,802.40**	39,021.32
80 - 84	14,627.34	**18,854.52**	23,912.33	12,283.47	**16,475.66**	21,624.18
85+	9,241.22	**12,481.48**	16,483.56	4,558.54	**7,036.59**	10,370.65
**Total**	208,219.96	**285,664.35**	389,692.53	251,077.34	**339,767.80**	455,406.97

2011-2012 HA SSI annual number of cases - McCabe 2						
Age group	Females			Males		
	2.5 %	Median	97.5 %	2.5 %	Median	97.5 %
0	2.79	**110.05**	600.88	33.14	**272.73**	963.43
01 - 04	0.00	**0.00**	1,353.06	10.11	**399.28**	2,183.05
05 - 09	235.80	**1,132.08**	3,174.53	0.00	**0.00**	1,735.62
10 - 14	0.00	**0.00**	1,630.80	9.95	**392.93**	2,104.89
15 - 19	115.97	**953.07**	3,332.20	0.00	**0.00**	1,146.05
20 - 24	118.95	**977.87**	3,426.72	59.67	**490.59**	1,719.76
25 - 29	0.00	**0.00**	1,870.21	44.71	**367.86**	1,296.47
30 - 34	0.00	**0.00**	2,064.37	104.42	**504.01**	1,445.05
35 - 39	502.38	**1,834.01**	4,614.92	326.24	**999.57**	2,299.30
40 - 44	1,003.27	**2,481.01**	5,043.79	599.39	**1,626.06**	3,499.97
45 - 49	1,567.65	**3,253.20**	5,925.12	903.30	**2,085.47**	4,079.98
50 - 54	2,085.07	**4,019.18**	6,968.71	1,459.99	**2,818.19**	4,899.05
55 - 59	3,527.67	**5,676.62**	8,623.85	4,998.42	**7,330.15**	10,350.41
60 - 64	2,946.05	**4,957.23**	7,799.20	7,167.38	**9,760.03**	12,964.88
65 - 69	5,344.36	**7,840.08**	11,075.74	7,403.09	**9,945.65**	13,061.83
70 - 74	5,285.84	**7,759.53**	10,972.83	9,263.95	**12,105.60**	15,525.19
75 - 79	5,868.79	**8,317.18**	11,429.22	7,332.66	**9,795.22**	12,805.27
80 - 84	4,036.43	**5,973.51**	8,508.62	4,545.52	**6,443.32**	8,857.02
85+	2,192.36	**3,538.64**	5,401.83	1,957.53	**3,200.14**	4,931.00
**Total**	34,833.39	**58,823.25**	103,816.61	46,219.46	**68,536.80**	105,868.21

2011-2012 HA SSI annual number of cases - McCabe 3						
Age group	Females			Males		
	2.5 %	Median	97.5 %	2.5 %	Median	97.5 %
0	2.29	**90.62**	481.03	0.00	**0.00**	374.23
01 - 04	0.00	**0.00**	835.37	0.00	**0.00**	1,190.55
05 - 09	0.00	**0.00**	965.09	0.00	**0.00**	1,088.98
10 - 14	8.53	**337.60**	1,209.32	0.00	**0.00**	910.93
15 - 19	0.00	**0.00**	1,170.06	0.00	**0.00**	868.27
20 - 24	0.00	**0.00**	1,464.92	0.00	**0.00**	599.29
25 - 29	0.00	**0.00**	2,289.41	2.67	**105.49**	555.99
30 - 34	11.46	**452.84**	2,398.55	32.81	**269.05**	926.80
35 - 39	82.72	**678.46**	2,341.03	0.00	**0.00**	599.52
40 - 44	0.00	**0.00**	945.35	0.00	**0.00**	848.07
45 - 49	7.10	**280.29**	1,535.66	428.63	**1,159.68**	2,479.36
50 - 54	171.60	**829.17**	2,387.76	873.44	**1,809.46**	3,284.37
55 - 59	511.51	**1,387.45**	2,985.16	1,237.79	**2,312.39**	3,916.47
60 - 64	473.77	**1,286.54**	2,776.10	811.86	**1,687.46**	3,083.13
65 - 69	457.03	**1,241.33**	2,679.82	739.67	**1,537.75**	2,810.81
70 - 74	817.12	**1,780.63**	3,355.33	1,113.50	**2,084.04**	3,541.55
75 - 79	1,145.05	**2,208.69**	3,834.43	596.16	**1,300.89**	2,458.21
80 - 84	567.86	**1,312.41**	2,573.50	369.52	**917.22**	1,880.87
85+	469.90	**1,086.98**	2,135.68	133.47	**489.28**	1,248.07
**Total**	4,725.93	**12,973.01**	38,363.57	6,339.51	**13,672.70**	32,665.47

Healthcare-associated urinary tract infection (HA UTI)

2011-2012 HA UTI annual number of cases - McCabe 1						
Age group	Females			Males		
	2.5 %	**Median**	97.5 %	2.5 %	**Median**	97.5 %
0	70.14	**579.12**	2,090.88	1,294.21	**2,829.37**	5,367.24
01 - 04	2,074.36	**5,647.59**	12,265.57	2,426.83	**6,608.34**	14,358.41
05 - 09	34.67	**1,369.25**	7,610.25	17.07	**674.34**	3,750.32
10 - 14	1,256.10	**4,605.88**	11,757.64	0.00	**0.00**	3,611.40
15 - 19	644.69	**2,364.80**	6,043.92	447.73	**2,169.90**	6,327.02
20 - 24	3,808.31	**8,322.44**	15,774.72	1,180.12	**3,631.47**	8,454.56
25 - 29	4,079.65	**8,917.80**	16,912.66	1,134.96	**3,089.97**	6,710.65
30 - 34	11,016.92	**18,899.63**	30,227.06	2,671.60	**5,564.85**	10,210.74
35 - 39	12,102.26	**19,072.95**	28,576.10	2,002.90	**4,978.16**	10,239.94
40 - 44	2,341.64	**5,117.65**	9,701.73	1,316.85	**3,586.40**	7,795.56
45 - 49	6,587.88	**11,515.30**	18,671.66	5,620.52	**9,474.40**	14,949.98
50 - 54	7,732.02	**11,935.51**	17,591.49	7,808.33	**12,306.16**	18,438.58
55 - 59	10,079.53	**14,923.66**	21,271.68	12,194.80	**17,302.45**	23,815.36
60 - 64	16,515.67	**22,531.12**	30,003.88	14,886.98	**20,240.63**	26,879.29
65 - 69	20,072.92	**26,402.44**	34,076.88	15,988.21	**21,384.56**	28,002.88
70 - 74	22,842.51	**29,268.42**	36,924.42	27,539.15	**34,968.46**	43,764.31
75 - 79	36,608.79	**44,442.50**	53,434.31	27,893.42	**35,604.45**	44,766.06
80 - 84	32,167.98	**38,907.61**	46,622.45	19,690.74	**25,267.56**	31,913.19
85+	41,569.46	**48,847.71**	57,011.35	17,082.51	**22,140.24**	28,204.61
**Total**	231,605.49	**323,671.38**	456,568.63	161,196.94	**231,821.70**	337,560.09

2011-2012 HA UTI annual number of cases - McCabe 2						
Age group	Females			Males		
	2.5 %	**Median**	97.5 %	2.5 %	**Median**	97.5 %
0	183.40	**667.39**	1,662.08	150.49	**547.79**	1,365.54
01 - 04	0.00	**0.00**	1,915.17	13.82	**546.11**	2,985.87
05 - 09	26.88	**1,061.75**	5,726.08	13.19	**521.02**	2,814.86
10 - 14	0.00	**0.00**	1,949.33	7.94	**313.70**	1,680.46
15 - 19	10.99	**434.32**	2,351.86	0.00	**0.00**	1,473.68
20 - 24	15.73	**621.40**	3,371.59	0.00	**0.00**	780.28
25 - 29	277.48	**1,336.89**	3,803.84	4.04	**159.40**	869.21
30 - 34	0.00	**0.00**	2,183.28	3.23	**127.64**	700.50
35 - 39	0.00	**0.00**	1,891.66	402.56	**1,090.30**	2,337.18
40 - 44	221.99	**1,073.77**	3,105.61	282.29	**1,032.93**	2,618.77
45 - 49	503.19	**1,366.73**	2,950.71	359.79	**1,105.65**	2,564.46
50 - 54	1,788.65	**3,569.65**	6,341.29	1,423.34	**2,844.31**	5,065.46
55 - 59	3,208.26	**5,394.22**	8,475.77	1,314.18	**2,627.85**	4,685.65
60 - 64	2,875.69	**4,923.46**	7,848.14	2,609.76	**4,264.74**	6,567.35
65 - 69	6,699.26	**9,897.60**	14,063.54	4,021.37	**5,992.20**	8,579.63
70 - 74	7,134.73	**10,214.72**	14,150.28	7,184.78	**9,904.79**	13,299.39
75 - 79	11,341.98	**14,894.32**	19,183.10	9,993.55	**13,031.33**	16,683.57
80 - 84	13,921.74	**17,509.61**	21,718.14	6,025.12	**8,416.47**	11,428.49
85+	16,332.92	**20,008.46**	24,247.09	7,754.32	**10,108.73**	12,937.38
**Total**	64,542.89	**92,974.29**	146,938.57	41,563.76	**62,634.96**	99,437.73

2011-2012 HA UTI annual number of cases - McCabe 3						
Age group	Females			Males		
	2.5 %	**Median**	97.5 %	2.5 %	**Median**	97.5 %
0	0.00	**0.00**	287.92	0.00	**0.00**	544.01
01 - 04	0.00	**0.00**	202.02	0.00	**0.00**	1,165.26
05 - 09	0.00	**0.00**	640.06	0.00	**0.00**	1,326.82
10 - 14	4.38	**173.42**	621.19	0.00	**0.00**	1,495.09
15 - 19	0.00	**0.00**	1,336.73	2.31	**91.29**	448.52
20 - 24	13.19	**521.27**	2,631.45	0.00	**0.00**	946.11
25 - 29	0.00	**0.00**	8,674.31	0.00	**0.00**	264.79
30 - 34	156.97	**1,285.18**	4,380.64	0.00	**0.00**	685.43
35 - 39	8.54	**337.50**	1,806.72	0.00	**0.00**	952.20
40 - 44	5.66	**223.51**	1,218.52	29.32	**241.20**	849.27
45 - 49	77.96	**641.95**	2,274.82	44.91	**370.16**	1,319.60
50 - 54	4.93	**194.66**	1,072.02	126.27	**610.96**	1,769.55
55 - 59	116.97	**565.93**	1,638.35	600.17	**1,487.82**	3,041.97
60 - 64	2,057.03	**3,737.75**	6,198.36	849.64	**1,766.00**	3,226.63
65 - 69	656.50	**1,626.83**	3,323.15	408.69	**1,014.15**	2,078.34
70 - 74	897.92	**1,730.13**	2,997.52	1,126.44	**2,172.91**	3,772.65
75 - 79	2,754.60	**4,373.56**	6,570.17	928.48	**1,855.19**	3,303.16
80 - 84	3,030.02	**4,615.22**	6,711.36	2,327.37	**3,578.25**	5,241.90
85+	3,881.45	**5,492.41**	7,531.44	2,115.77	**3,286.99**	4,856.33
**Total**	13,666.13	**25,519.29**	60,116.77	8,559.37	**16,474.92**	37,287.62
