# Supplementary material for: Burden of Six Healthcare-Associated Infections on European Population Health: Estimating Incidence-Based Disability-Adjusted Life Years through a Population Prevalence-Based Modelling Study
Source: PLoS Med. 2016 Oct 18;13(10):e1002150. doi: 10.1371/journal.pmed.1002150 (PMC5068791; doi:10.1371/journal.pmed.1002150)
Supplement: S1 Models — (PDF) [file pmed.1002150.s002.pdf]

Burden of Communicable Diseases in Europe Project

Office of the Chief Scientist Unit

Supplementary Information 1
Healthcare-associated infections
Disease outcome trees

Produced by Alessandro Cassini, Diamantis Plachouras and Carl Suetens (ECDC)

Healthcare-associated Clostridium difficile infection (HA CDI)

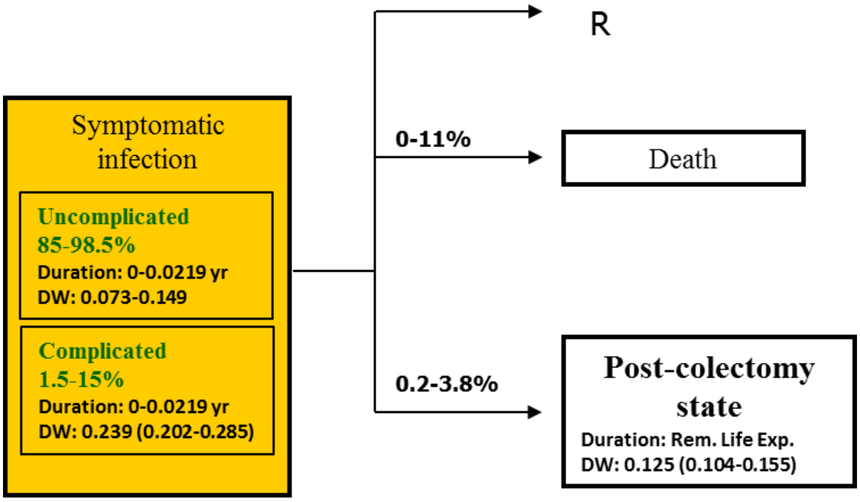

Risk of symptomatic infection and duration of disease

Clostridium difficile infection (CDI) is defined by a positive laboratory test for C. difficile (C. difficile toxin A and/or/B or positive culture for toxin-producing C. difficile or cell cytotoxicity assay or PCR) or pseudomembranous colitis, identified through colonoscopy or histopathology or autopsy (ECDC, 2012).

We performed a search for systematic reviews and two additional searches for primary articles on the outcomes 'length of hospital stay' (LOS), 'death' and 'sepsis'. To differentiate between health states (i.e. mild and moderate vs. severe CDI), data on the severity of CDI were collected in a non-systematic fashion.

For the outcomes 'LOS', 'death' and 'sepsis' we extracted risk differences, whereas for the outcomes 'recurrence' and 'post-colectomy state' absolute risks were used. In the case of the outcome 'post-colectomy state' this approach was chosen assuming that colectomy is a rare event in patients without CDI.

The outcome tree was constructed starting from acute CDI that was divided into two different health states: mild and moderate versus severe CDI, the latter representing 1.5–15% of CDI episodes (HPSC, 2012; Kanerva, 2013; Bauer, 2011; CDAD KISS, 2012). The duration of the acute disease was described by the attributable LOS, a surrogate marker for short-term complications such as ICU stay or toxic megacolon. The attributable LOS ranged from zero to eight days (Campbell, 2013; Dodek, 2013; Kyne, 2002; Song, 2008; Tabak, 2013; Vonberg, 2008). The transitional probability for the outcome 'death' was expressed by the overall attributable mortality (including death after colectomy) of 0–11% (lower boundary of -3% was set to zero) (Dodek, 2013; Song, 2008; Tabak, 2013; Oake, 2010).

It was not possible to make a quantitative analysis of the transitional probabilities for the outcomes 'death' and 'LOS' by pooling the data. This was due to (a) differing methods of adjustment by design or analysis (matching, propensity score matching, multivariate analysis) across the studies, (b) adjustment factors not being comparable between the studies and (c) variance estimates not being provided for all studies.

The study by Pépin et al (2005), conducted during an epidemic caused by C. difficile PCR ribotype 027 reported the highest estimates for LOS and attributable mortality. As the study setting was not representative of a setting where CDIs generally occur, after consultation with the experts we decided not to include the study.

The transitional probabilities for the outcome 'post-colectomy state' ranged between 0.2 and 3.8% (Bhangu, 2012). The duration of this outcome was lifelong.

Calculation of a point estimate by pooling incidences did not seem appropriate without knowing the baseline risks for colectomy within the studies. In the majority of articles, the characteristics of the study population only referred to patients with colectomy because the selected studies focused on outcomes after colectomy.

Since the systematic search for sepsis did not reveal any eligible studies, this outcome was omitted.

From a clinical point of view, recurrence is a major issue in patients with CDI. Nevertheless, inclusion of recurrence (e.g. by introducing one or more loops in the outcome tree) may have led to double counting of cases, and subsequently to an overestimation of the burden of disease, depending on how the issue of recurrence was dealt with by the prevalence data fitted into the tree. Therefore, we chose not to include recurrence in the outcome tree.

Model input summary

Table 1. Transition probabilities used in the outcome tree

Health outcome (Health state)	Distribution of health states in health outcome	Transition probability	Source/assumption
Symptomatic disease			HPSC, 2012; Kanerva, 2013; Bauer, 2011;

Uncomplicated<br><br>Complicated	85-98.5%<br><br>1.5-15%		CDAD KISS, 2012
Fatal cases following symptomatic infection		0-11%	Dodek, 2013; Song, 2008; Tabak, 2013; Oake, 2010
Post-colectomy state		0.2-3.8%	Bhangu, 2012

Table 2. Disability weights and duration

Health outcome (Health state)	Disability Weight (DW) (Haagsma, 2015)		Duration	
	DW	Label	In years	Source/assumption
Symptomatic disease			0-0.0219	

Uncomplicated<br><br>Complicated	0.073-0.149<br><br>0.239 (0.202-0.285)	Diarrhoea, from mild to moderate<br><br>Diarrhoea, severe		Campbell, 2013; Dodek, 2013; Kyne, 2002; Song, 2008; Tabak, 2013;<br>Vonberg, 2008
Post-colectomy state	0.125 (0.104-0.155)	Stoma	Remaining Life Expectancy	

References

BHANGU, A., NEPOGODIEV, D., GUPTA, A., TORRANCE, A., SINGH, P. & WEST MIDLANDS RESEARCH, C. 2012. Systematic review and meta- analysis of outcomes following emergency surgery for Clostridium difficile colitis. Br J Surg, 99, 1501-13.

BAUER, M. P., NOTERMANS, D. W., VAN BENTHEM, B. H., BRAZIER, J. S., WILCOX, M. H., RUPNIK, M., MONNET, D. L., VAN DISSEL, J. T., KUIJPER, E. J. & GROUP, E. S. 2011. Clostridium difficile infection in Europe: a hospital-based survey. Lancet, 377, 63-73.

CAMPBELL, R., DEAN, B., NATHANSON, B., HAIDAR, T., STRAUSS, M., THOMAS, S., CAMPBELL, R., DEAN, B., NATHANSON, B., HAIDAR, T., STRAUSS, M. & THOMAS, S. 2013. Length of stay and hospital costs among high-risk patients with hospital-origin Clostridium difficile-associated diarrhea. Journal of medical economics, 16, 440-448.

CDAD. 2012. CDAD KISS reference data 2012. Available: http://www.nrz-hygiene.de/surveillance/kiss/cdad-kiss/.

DODEK, P. M., NORENA, M., AYAS, N. T., ROMNEY, M. & WONG, H. 2013. Length of stay and mortality due to Clostridium difficile infection acquired in the intensive care unit. Journal of critical care, 28, 335-40.

Haagsma JA, Maertens de Noordhout C, Polinder S, Vos T, Havelaar AH, Cassini A, Devleesschauwer B, Kretzschmar ME, Speybroeck N, Salomon JA. Assessing disability weights based on the responses of 30,660 people from four European countries. Population Health Metrics 2015; 13: 10

HEALTH PROTECTION SURVEILLANCE CENTRE (HPSC), A. R. 2012. Health Protection Surveillance Centre (HPSC), Annual Report 2012. Available: http://www.hpsc.ie/A-Z/Gastroenteric/Clostridiumdifficile/CdifficileSurveillance/AnnualReports/File,14557,en.pdf

KANERVA, M., MENTULA, S., VIROLAINEN-JULKUNEN, A., KARKI, T., MOTTONEN, T. & LYYTIKAINEN, O. 2013. Reduction in Clostridium difficile infections in Finland, 2008-2010. J Hosp Infect, 83, 127-31.

KYNE, L., HAMEL, M. B., POLAVARAM, R. & KELLY, C. P. 2002. Health care costs and mortality associated with nosocomial diarrhea due to Clostridium difficile. Clinical infectious diseases : an official publication of the Infectious Diseases Society of America, 34, 346-53.

OAKE, N., TALJAARD, M., VAN WALRAVEN, C., WILSON, K., ROTH, V. & FORSTER, A. J. 2010. The effect of hospital-acquired Clostridium difficile infection on in-hospital mortality. Arch Intern Med, 170, 1804-10.

SONG, X., BARTLETT, J. G., SPECK, K., NAEGELI, A., CARROLL, K. & PERL, T. M. 2008. Rising economic impact of clostridium difficile-associated disease in adult hospitalized patient population. Infection control and hospital epidemiology : the official journal of the Society of Hospital Epidemiologists of America, 29, 823-8.

TABAK, Y. P., ZILBERBERG, M. D., JOHANNES, R. S., SUN, X., MCDONALD, L. C., TABAK, Y. P., ZILBERBERG, M. D., JOHANNES, R. S., SUN, X. & MCDONALD, L. C. 2013. Attributable burden of hospital-onset Clostridium difficile infection: A propensity score matching study. Infection Control and Hospital Epidemiology, 34, 588-596.

VONBERG, R. P., REICHARDT, C., BEHNKE, M., SCHWAB, F., ZINDLER, S. & GASTMEIER, P. 2008. Costs of nosocomial Clostridium difficile- associated diarrhoea. The Journal of hospital infection, 70, 15-20.

Healthcare-associated pneumonia (HAP)

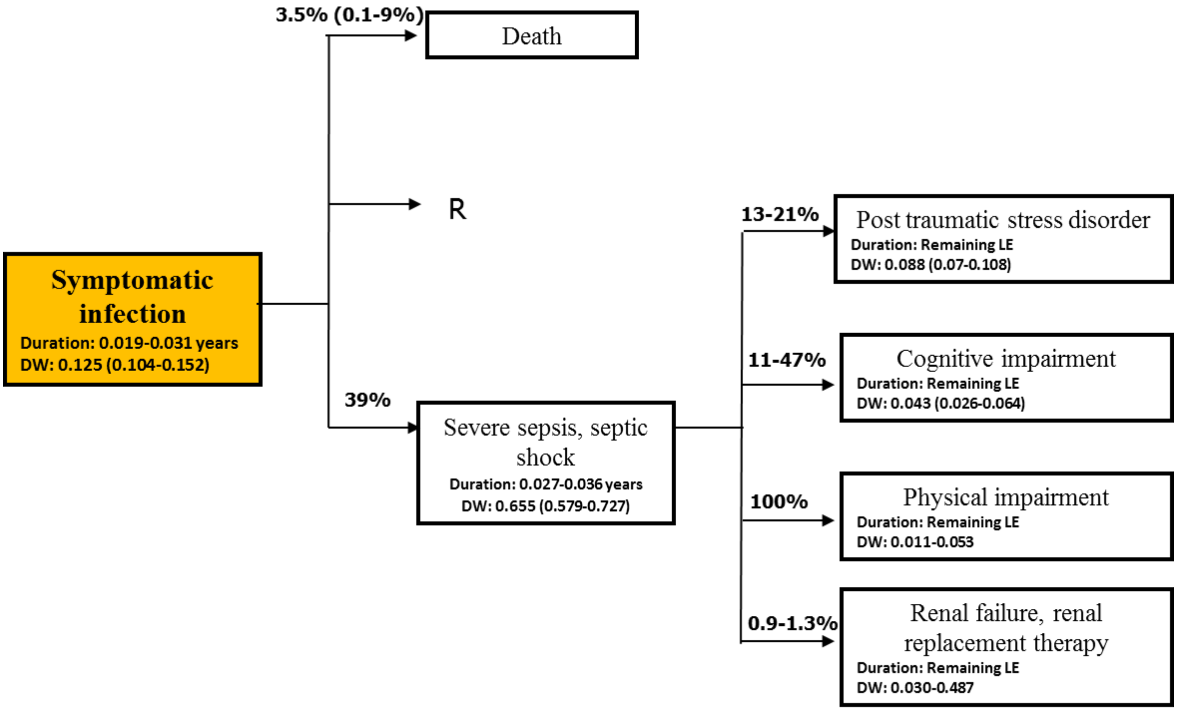

Risk of symptomatic infection and duration of disease

Healthcare-associated pneumonia (HAP) was defined in accordance with the ECDC case definition (ECDC, 2012). A systematic review of the literature was performed to estimate the sequelae and the probability of developing these sequelae following HAP. This review took into consideration the role of co-morbidities by estimating the attributable impact of HAP: attributable mortality, attributable risk of developing sequelae and its duration (effect on the length of hospital stay).

The extracted literature was limited to ventilator-associated pneumonia (VAP) because no specific literature was found for the more general search terms 'pneumonia' and 'lower respiratory tract infection'.

Attributable ICU-mortality due to VAP varied from 0.1% to 9% based on the studies by Aybar Türkoglu et al. (2008) and Rello et al. (2002). The report on surveillance of healthcare-associated infections in intensive care units in Europe, 2008-2012, stemming from the HAI-Net ICU surveillance (ECDC, 2016), found an overall attributable case fatality proportion for pneumonia patients of 3.5% that was used as the median estimate in our model.

Attributable length of stay in an intensive care unit (ICU) due to VAP varied from 2.03 to 7 days whereas attributable length of hospital stay following VAP varied from 7 to 11.5 days. The latter range is included in the outcome tree (Aybar-Türkoglu, 2008; Rello, 2002).

Sepsis and acute respiratory distress syndrome (ARDS) are generally considered to be frequent consequences of VAP. However, only one study provided data on the transitional probability which amounted to 39% (proportion of patients suffering from severe sepsis and/or septic shock) for sepsis/ARDS as a health consequence of VAP (Damas, 2011). Duration of severe sepsis or septic shock was 9.9–13 days (Olaechea, 2013; Renaud, 2001). The long-term health outcomes following sepsis and ARDS were taken from the model developed for healthcare-associated primary bloodstream infection.

Model input summary

Table 1. Transition probabilities used in the outcome tree

Health outcome (Health state)	Distribution of health states in health outcome	Transition probability	Source/assumption
Fatal cases following symptomatic infection		3.5% (0.1-9)%	Aybar Türkoglu, 2008; Rello, 2002
Severe sepsis or septic shock		39%	Damas, 2011
Post-traumatic stress disorder (PTSD) following severe sepsis or septic shock		13-21%	Kessler, 1995; Deja, 2006; Schelling, 1998; Stoll, 1999; Kapfhammer, 2004; Hopkins, 2005
Cognitive impairment following severe sepsis or		11-47%	Hopkins, 2005; Iwashyna, 2010

septic shock			
Physical impairment following severe sepsis or septic shock		100%	Hopkins, 2005; Herridge, 2003; Hofhuis, 2008
Renal failure and renal replacement therapy following severe sepsis or septic shock		0.9-1.3%	Gallagher, 2014; Wisplinghoff, 2004

Table 2. Disability weights and duration

Health outcome (Health state)	Disability Weight (DW) (Haagsma, 2015)		Duration	
	DW	Label	In years	Source/assumption
Symptomatic infection	0.125 (0.104-0.152)	Infectious disease, acute episode, severe	0.019-0.031	Aybar Türkoglu, 2008; Rello, 2002
Severe sepsis or septic shock	0.655 (0.579-0.727)	Intensive care unit admission	0.027-0.036	Olaechea, 2013; Renaud, 2001
Post-traumatic stress disorder (PTSD) following severe sepsis or septic shock	0.088 (0.07-0.108)	Subacute sclerosing panencephalitis – phase 1<br><br>(chosen according to best fitting description)	Remaining life expectancy	

Cognitive impairment following severe sepsis or septic shock	0.043 (0.026-0.064)	Intellectual disability, mild	Remaining life expectancy	
Physical impairment following severe sepsis or septic shock	0.011-0.053	Range between motor impairment, mild and motor impairment, moderate	Remaining life expectancy	
Renal failure and renal replacement therapy following severe sepsis or septic shock	0.030-0.487	Range between end-stage renal disease, on dialysis and end-stage renal disease, with kidney transplant	Remaining life expectancy	

References

Aybar Türkoglu, m. & Topeli Iskit, A. 2008. Ventilator-associated pneumonia caused by high risk microorganisms: a matched case-control study. Tüberküloz ve toraks, 56, 139-49.

Bercault, N. & Boulain, T. 2001. Mortality rate attributable to ventilator-associated nosocomial pneumonia in an adult intensive care unit: a prospective case-control study. Critical care medicine, 29, 2303-9.

Damas, P., Layios, N., Seidel, l., Nys, M., Melin, P. & Ledoux, D. 2011. Severity of ICU-acquired pneumonia according to infectious microorganisms. Intensive care medicine, 37, 1128-35.

European Centre for Disease Prevention and Control. Point prevalence survey of healthcare-associated infections and antimicrobial use in European acute care hospitals – protocol version 4.3. Stockholm: ECDC; 2012. Available at: http://ecdc.europa.eu/en/publications/Publications/0512-TED-PPS-HAI-antimicrobial-use-protocol.pdf

European Centre for Disease Prevention and Control. Surveillance of healthcare-associated infections in intensive care units in Europe, 2008-2012. Stockholm: ECDC; 2016. In press

Gallagher, M., Cass, A., bellomo, R., Finfer, S., Gattas, D., Lee, J., Lo, S., Mcguinness, S., Myburgh, j., Parke, R. & Rajbhandari, D. 2014. Long-Term Survival and Dialysis Dependency Following Acute Kidney Injury in Intensive Care: Extended Follow-up of a Randomized Controlled Trial. PLoS Med, 11, e1001601.

Haagsma JA, Maertens de Noordhout C, Polinder S, Vos T, Havelaar AH, Cassini A, Devleesschauwer B, Kretzschmar ME, Speybroeck N, Salomon JA. Assessing disability weights based on the responses of 30,660 people from four European countries. Population Health Metrics 2015; 13: 10

Herridge, M. S., Cheung, A. M., tansey, C. M., Matte-martyn, A., Diaz-granados, N., Al-saidi, F., Cooper, A. b., Guest, C. B., Mazer, C. D., Mehta, S., Stewart, T. E., Barr, A., Cook, D. & Slutsky, A. S. 2003. One-Year Outcomes in Survivors of the Acute Respiratory Distress Syndrome. New England Journal of Medicine, 348, 683-693.

Hofhuis, J. G., Spronk, P. E., van Stel, H. F., Schrijvers, A. J., Rommes, J. H. & Bakker, J. 2008. The impact of severe sepsis on health-related quality of life: a long-term follow-up study. Anesth Analg, 107, 1957-64.

Hopkins, R. O., Weaver, L. K., collingridge, D., Parkinson, R. B., Chan, K. J. & Orme, J. F., Jr. 2005. Two-year cognitive, emotional, and quality-of- life outcomes in acute respiratory distress syndrome. Am J Respir Crit Care Med, 171, 340-7

Iwashyna, T. J., Ely, E. W., smith, D. M. & Langa, K. M. 2010. Long-term cognitive impairment and functional disability among survivors of severe sepsis. JAMA, 304, 1787-94.

Kessler, R. C., Sonnega, A., bromet, E., Hughes, M. & Nelson, C. B. 1995. Posttraumatic stress disorder in the National Comorbidity Survey. Arch Gen Psychiatry, 52, 1048-60.

Olaechea, P. M., Palomar, M., alvarez-lerma, F., Otal, J. J., Insausti, J., Lopez-pueyo, M. J. & Group, e.-h. 2013. Morbidity and mortality associated with primary and catheter-related bloodstream infections in critically ill patients. Rev Esp Quimioter, 26, 21-9.

Melsen, W. G., Rovers, M. M., Groenwold, r. H., Bergmans, D. C., Camus, C., Bauer, T. T., Hanisch, E. W., Klarin, B., koeman, M., Krueger, W. A., Lacherade, J. C., Lorente, L., Memish, Z. A., morrow, L. E., Nardi, G., Van Nieuwenhoven, C. A., O'keefe, G. E., Nakos, G., scannapieco, F. A., Seguin, P., Staudinger, T., Topeli, A., Ferrer, M. & bonten, M. J. 2013. Attributable mortality of ventilator-associated pneumonia: a meta-analysis of individual patient data from randomised prevention studies. The Lancet infectious diseases, 13, 665-71.

Rello, J., Ollendorf, D. A., oster, G., Vera-llonch, M., Bellm, L., Redman, R., Kollef, M. H. & Group, v. A. P. O. S. A. 2002. Epidemiology and outcomes of ventilator-associated pneumonia in a large US database. Chest, 122, 2115-21.

Renaud, B., Brun-buisson, C. & Group, I. C.-b. S. 2001. Outcomes of primary and catheter-related bacteremia. A cohort and case-control study in critically ill patients. Am J Respir Crit Care Med, 163, 1584-90.

Healthcare-associated neonatal sepsis

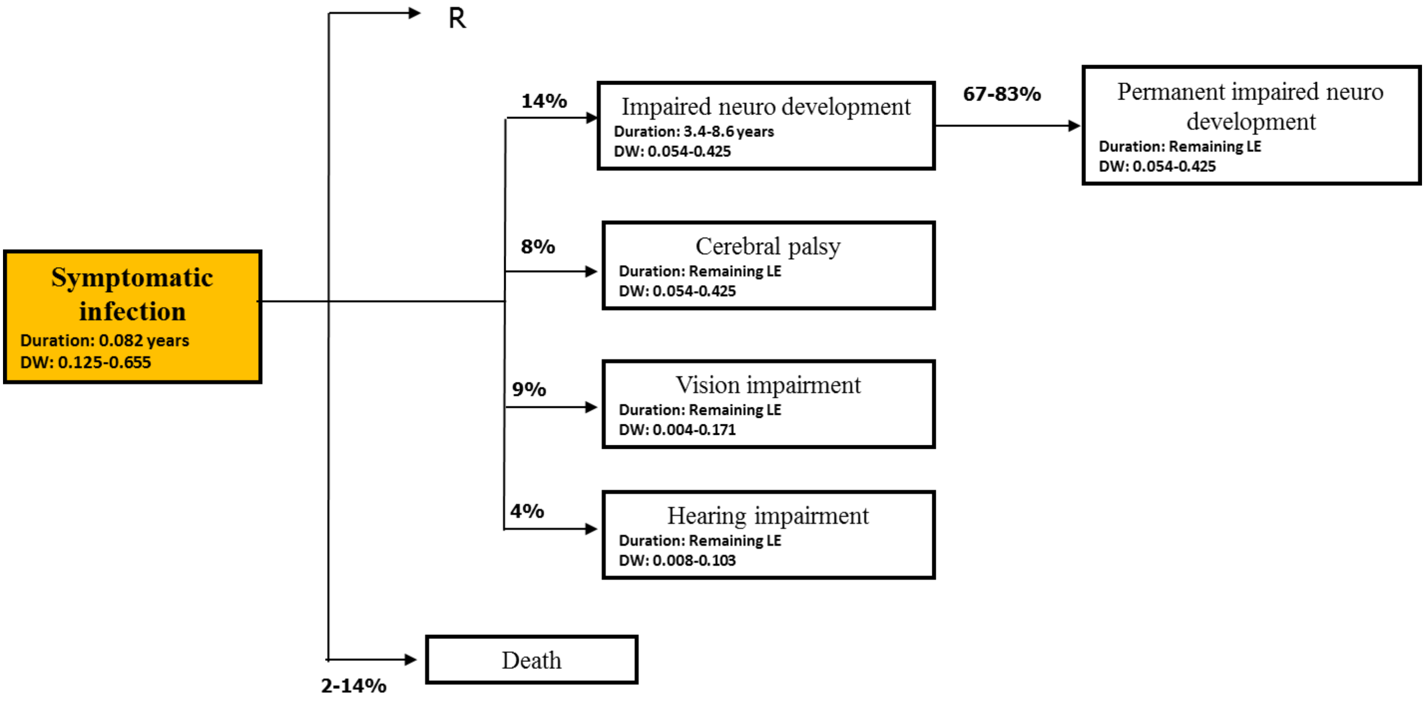

Risk of symptomatic infection and duration of disease

Neonatal sepsis is characterised in accordance with the definition set out in ECDC's point prevalence survey of healthcare-associated infections and antimicrobial use in European acute care hospitals with the code NEO-LCBI – laboratory-confirmed bloodstream infection in neonates (ECDC, 2012).

The entire disease model was based on the systematic review of the natural history of neonatal sepsis, conducted for the purpose of estimating burden of disease by Haller et al. (2015, in press). Given the lack of studies referring to all sepsis in term infants, this outcome tree represents very low weight birth (VLBW) infants. This must be taken into account when inputting the number of cases.

Model input summary

Table 1. Transition probabilities used in the outcome tree

Health outcome (Health state)	Distribution of health states in health outcome	Transition probability	Source/assumption
Fatal cases following symptomatic infection		2-14%	Haller, 2015
Vision impairment		9%	Haller, 2015
Hearing impairment		4%	Haller, 2015
Cerebral palsy		8%	Haller, 2015
Impaired neuro development		14%	Haller, 2015
Permanent impaired neuro development		67-83%	Haller, 2015

Table 2. Disability weights and duration

Health outcome (Health state)	Disability Weight (DW) (Haagsma, 2015)		Duration	
	DW	Label	In years	Source/assumption
Symptomatic infection	0.125-0.655	Range between Infectious disease, acute episode,	0.082	Haller, 2015

		severe and<br><br>Intensive care unit admission DWs		
Vision impairment	0.004-0.171	From lowest to highest vision impairment related DWs	Remaining life expectancy	Haller, 2015
Hearing impairment	0.008-0.103	From lowest to highest hearing loss related DWs	Remaining life expectancy	Haller, 2015
Cerebral palsy	0.054-0.425	From lowest to highest Motor plus cognitive impairments related DWs	Remaining life expectancy	Haller, 2015
Impaired neuro development	0.054-0.425	From lowest to highest Motor plus cognitive impairments related DWs	3.4-8.6	Haller, 2015
Permanent impaired neuro development	0.054-0.425	From lowest to highest Motor plus cognitive impairments related DWs	Remaining life expectancy	Haller, 2015

References

European Centre for Disease Prevention and Control. Point prevalence survey of healthcare-associated infections and antimicrobial use in European acute care hospitals – protocol version 4.3. Stockholm: ECDC; 2012. Available at: http://ecdc.europa.eu/en/publications/Publications/0512-TED-PPS-HAI-antimicrobial-use-protocol.pdf

Haagsma JA, Maertens de Noordhout C, Polinder S, Vos T, Havelaar AH, Cassini A, Devleesschauwer B, Kretzschmar ME, Speybroeck N, Salomon JA. Assessing disability weights based on the responses of 30,660 people from four European countries. Population Health Metrics 2015; 13: 10

Haller S, Deindl P, Cassini A, Suetens C, Zingg W, Abu Sin M, Velasco E, Weiß B, Ducomble T, Sixtensson M, Eckmanns T, Thomas H. Evidence- based outcome tree for neurological sequelae of sepsis in very low birth weight infants. 2015, Submitted

Healthcare-associated Primary blood-stream infections (P-BSI)

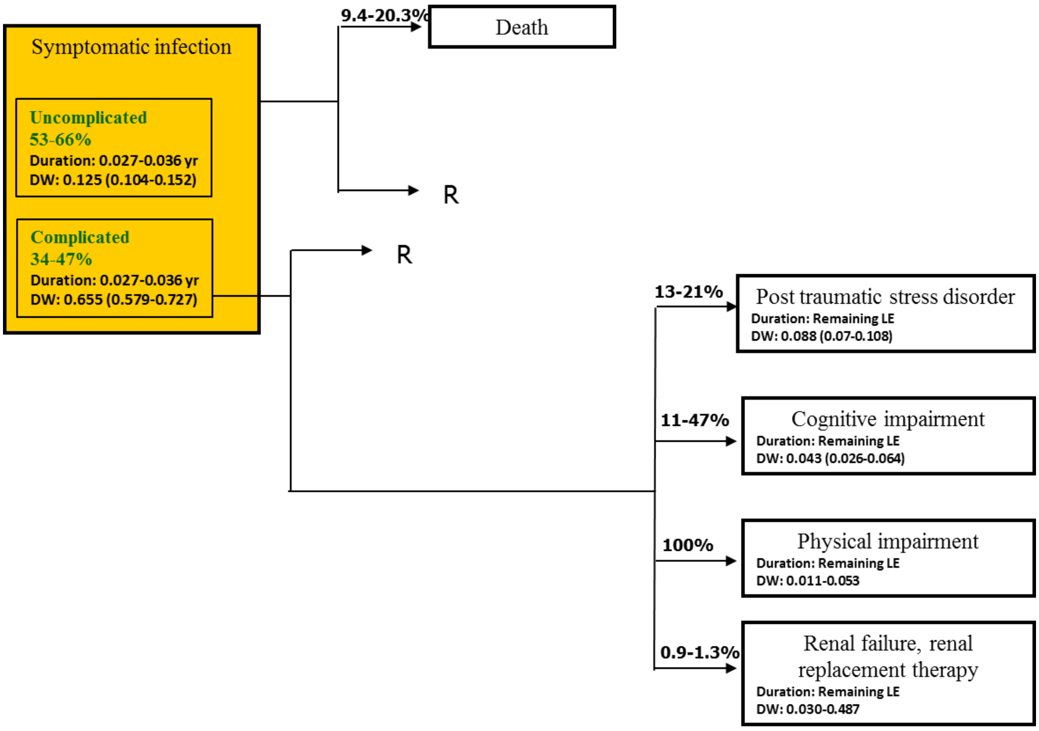

Risk of symptomatic infection and duration of disease

A healthcare-associated primary bloodstream infection (HA primary BSI) in adults was defined in accordance with the ECDC case definition (ECDC, 2012).

The distribution into complicated vs. uncomplicated cases resulting from an initial symptomatic infection was 34–47% vs. 53–66% (Engel, 2007; Pittet, 1995; Rangel-Frausto, 1995).

The disability weights for hospitalisation in an intensive care unit (ICU) and for complicated sepsis already included the disease weight for short-term complications. Therefore, details of the risk differences are only provided for information purposes since they are not required for the outcome tree. Short-term complications include renal failure with renal replacement therapy (17–23%) (Gallagher, 2014; Oppert, 2008; Wisplinghoff, 2004), critical illness myopathy/polyneuropathy (50–75%) (Khan, 2006; Garnacho-Montero, 2001) and sepsis-associated encephalopathy (50–62%) (Eidelman, 1996).

For some of the long-term complications, we did not identify any relevant literature for cases with a health status of sepsis or BSI, although information was found for Acute Respiratory Distress Syndrome (ARDS). As ARDS frequently occurs following severe sepsis, we agreed with the clinical experts that ARDS could be used as a proxy for complicated sepsis.

Mortality was set to 9.4–20.3% (Renaud, 2001; Olaechea, 2013) and length of hospital stay) to 10–13 days (Renaud, 2001; Olaechea, 2013).

Risk of complications

Post-traumatic stress disorder (PTSD) is a significant sequelae of severe sepsis/septic shock. Given a population prevalence of 7.8% (Kessler, 1995), the resulting risk of developing PTSD was set at 13–21% (Deja, 2006; Schelling, 1998; Stoll, 1999; Kapfhammer, 2004; Hopkins, 2005).

Two studies were identified that reported on the risk of developing cognitive impairment following BSI, but the reported values differed significantly: 11% for Iwashyna et al. (2010) and 47% for Hopkins et al. (2005) . Major methodological differences included different ascertainment methods and study participants' health states at the inclusion phase.

A certain degree of physical impairment was reported in all patients during follow-up after severe sepsis/septic shock (Hopkins, 2005; Herridge, 2003; Hofhuis, 2008).

We assumed that the risk of chronic/long-term renal replacement therapy (RRT) was the same for all instances of acute renal failure, irrespective of cause. A total of 8% of BSI patients needed RRT at the onset of BSI (Wisplinghoff, 2004). In accordance with the American College of Chest Physicians/Society of Critical Care Medicine (ACCP/SCCM) definition, we classified BSI patients with acute renal failure as "complicated sepsis". Thus, the percentage of BSI patients needing RRT (8%) was weighted with severity proportions (complicated sepsis in 33.7–47.1%) and a range of 16.6–23.2% was estimated for patients with complicated sepsis that require RRT at

the onset of BSI. In conclusion, 16.6–23.2% of all patients with complicated sepsis patients were estimated to require RRT in the short-term and of those 5.4% were estimated to require long-term maintenance RRT. Assuming a background risk of zero, the risk of requiring long-term RRT after severe sepsis was 0.9–1.3%.

Model input summary

Table 1. Transition probabilities used in the outcome tree

Health outcome (Health state)	Distribution of health states in health outcome	Transition probability	Source/assumption
Symptomatic infection			Engel, 2007; Pittet, 1995; Rangel-Frausto, 1995
Uncomplicated	53-66%		
Complicated	34-47%		
Fatal cases following symptomatic infection		9.4-20.3%	Renaud, 2001; Olaechea, 2013
Post-traumatic stress disorder (PTSD) following severe sepsis or septic shock		13-21%	Kessler, 1995; Deja, 2006; Schelling, 1998; Stoll, 1999; Kapfhammer, 2004; Hopkins, 2005
Cognitive impairment following severe sepsis or septic shock		11-47%	Hopkins, 2005; Iwashyna, 2010
Physical impairment following severe sepsis or septic shock		100%	Hopkins, 2005; Herridge, 2003; Hofhuis, 2008
Renal failure and renal replacement therapy following severe sepsis or septic shock		0.9-1.3%	Gallagher, 2014; Wisplinghoff, 2004

Table 2. Disability weights and duration

Health outcome (Health state)	Disability Weight (DW) (Haagsma, 2015)		Duration	
	DW	Label	In years	Source/assumption
Symptomatic infection			0.027-0.036	Renaud, 2001; Olaechea, 2013
Uncomplicated	0.125 (0.104-0.152)	Infectious disease, acute episode, severe		
Complicated	0.655 (0.579-0.727)	Intensive care unit admission		
Post-traumatic stress disorder (PTSD) following severe sepsis or septic shock	0.088 (0.07-0.108)	Subacute sclerosing panencephalitis – phase 1<br>(chosen according to best fitting description)	Remaining life expectancy	
Cognitive impairment following severe sepsis or septic shock	0.043 (0.026-0.064)	Intellectual disability, mild	Remaining life expectancy	
Physical impairment following severe sepsis or septic shock	0.011-0.053	Range between Motor impairment, mild and Motor impairment, moderate	Remaining life expectancy	
Renal failure and renal replacement therapy following severe sepsis or septic shock	0.030-0.487	Range between End-stage renal disease, on dialysis and End-stage renal disease, with kidney transplant	Remaining life expectancy	

References

DEJA, M., DENKE, C., WEBER-CARSTENS, S., SCHRODER, J., PILLE, C. E., HOKEMA, F., FALKE, K. J. & KAISERS, U. 2006. Social support during intensive care unit stay might improve mental impairment and consequently health-related quality of life in survivors of severe acute respiratory distress syndrome. Crit Care, 10, R147.

Eidelman LA, Putterman D, Putterman C, Sprung CL. The spectrum of septic encephalopathy. Definitions, etiologies, and mortalities. JAMA. 1996;275(6):470-3.

Engel C, Brunkhorst FM, Bone HG, Brunkhorst R, Gerlach H, Grond S, et al. Epidemiology of sepsis in Germany: results from a national prospective multicenter study. Intensive Care Med. 2007;33(4):606-18.

European Centre for Disease Prevention and Control. Point prevalence survey of healthcare-associated infections and antimicrobial use in European acute care hospitals – protocol version 4.3. Stockholm: ECDC; 2012.

Gallagher M, Cass A, Bellomo R, Finfer S, Gattas D, Lee J, et al. Long-Term Survival and Dialysis Dependency Following Acute Kidney Injury in Intensive Care: Extended Follow-up of a Randomized Controlled Trial. PLoS Med. 2014;11(2):e1001601

GARNACHO-MONTERO, J., MADRAZO-OSUNA, J., GARCIA-GARMENDIA, J. L., ORTIZ-LEYBA, C., JIMENEZ-JIMENEZ, F. J., BARRERO-ALMODOVAR, A., GARNACHO-MONTERO, M. C. & MOYANO-DEL-ESTAD, M. R. 2001. Critical illness polyneuropathy: risk factors and clinical consequences. A cohort study in septic patients. Intensive Care Med, 27, 1288-96.

Haagsma JA, Maertens de Noordhout C, Polinder S, Vos T, Havelaar AH, Cassini A, Devleesschauwer B, Kretzschmar ME, Speybroeck N, Salomon JA. Assessing disability weights based on the responses of 30,660 people from four European countries. Population Health Metrics 2015; 13: 10

HERRIDGE, M. S., CHEUNG, A. M., TANSEY, C. M., MATTE-MARTYN, A., DIAZ-GRANADOS, N., AL-SAIDI, F., COOPER, A. B., GUEST, C. B., MAZER, C. D., MEHTA, S., STEWART, T. E., BARR, A., COOK, D. & SLUTSKY, A. S. 2003. One-Year Outcomes in Survivors of the Acute Respiratory Distress Syndrome. New England Journal of Medicine, 348, 683-693.

HOFHUIS, J. G., SPRONK, P. E., VAN STEL, H. F., SCHRIJVERS, A. J., ROMMES, J. H. & BAKKER, J. 2008. The impact of severe sepsis on health- related quality of life: a long-term follow-up study. Anesth Analg, 107, 1957-64.

HOPKINS, R. O., WEAVER, L. K., COLLINGRIDGE, D., PARKINSON, R. B., CHAN, K. J. & ORME, J. F., JR. 2005. Two-year cognitive, emotional, and quality-of-life outcomes in acute respiratory distress syndrome. Am J Respir Crit Care Med, 171, 340-7

IWASHYNA, T. J., ELY, E. W., SMITH, D. M. & LANGA, K. M. 2010. Long-term cognitive impairment and functional disability among survivors of severe sepsis. JAMA, 304, 1787-94.

KAPFHAMMER, H. P., ROTHENHAUSLER, H. B., KRAUSENECK, T., STOLL, C. & SCHELLING, G. 2004. Posttraumatic stress disorder and health- related quality of life in long-term survivors of acute respiratory distress syndrome. Am J Psychiatry, 161, 45-52.

KHAN, J., HARRISON, T. B., RICH, M. M. & MOSS, M. 2006. Early development of critical illness myopathy and neuropathy in patients with severe sepsis. Neurology, 67, 1421-5.

Olaechea PM, Palomar M, Alvarez-Lerma F, Otal JJ, Insausti J, Lopez-Pueyo MJ, et al. Morbidity and mortality associated with primary and catheter-related bloodstream infections in critically ill patients. Rev Esp Quimioter. 2013;26(1):21-9.

Oppert M, Engel C, Brunkhorst FM, Bogatsch H, Reinhart K, Frei U, et al. Acute renal failure in patients with severe sepsis and septic shock--a significant independent risk factor for mortality: results from the German Prevalence Study. Nephrol Dial Transplant. 2008;23(3):904-9.

Pittet D, Tarara D, Wenzel RP. Nosocomial bloodstream infection in critically ill patients. Excess length of stay, extra costs, and attributable mortality. JAMA : the journal of the American Medical Association. 1994;271(20):1598-601.

Renaud B, Brun-Buisson C, Group IC-BS. Outcomes of primary and catheter-related bacteremia. A cohort and case-control study in critically ill patients. Am J Respir Crit Care Med. 2001;163(7):1584-90.

Rangel-Frausto MS, Pittet D, Costigan M, Hwang T, Davis CS, Wenzel RP. The natural history of the systemic inflammatory response syndrome (SIRS). A prospective study. Jama. 1995;273(2):117-23.

SCHELLING, G., STOLL, C., HALLER, M., BRIEGEL, J., MANERT, W., HUMMEL, T., LENHART, A., HEYDUCK, M., POLASEK, J., MEIER, M., PREUSS, U., BULLINGER, M., SCHUFFEL, W. & PETER, K. 1998. Health-related quality of life and posttraumatic stress disorder in survivors of the acute respiratory distress syndrome. Crit Care Med, 26, 651-9.

STOLL, B. J., HANSEN, N. I., ADAMS-CHAPMAN, I., FANAROFF, A. A., HINTZ, S. R., VOHR, B. & HIGGINS, R. D. 2004. Neurodevelopmental and growth impairment among extremely low-birth-weight infants with neonatal infection. Jama, 292, 2357-65.

Wisplinghoff H, Bischoff T, Tallent SM, Seifert H, Wenzel RP, Edmond MB. Nosocomial bloodstream infections in US hospitals: analysis of 24,179 cases from a prospective nationwide surveillance study. Clinical infectious diseases : an official publication of the Infectious Diseases Society of America. 2004;39(3):309-17.

Healthcare-associated surgical site infection (HA SSI)

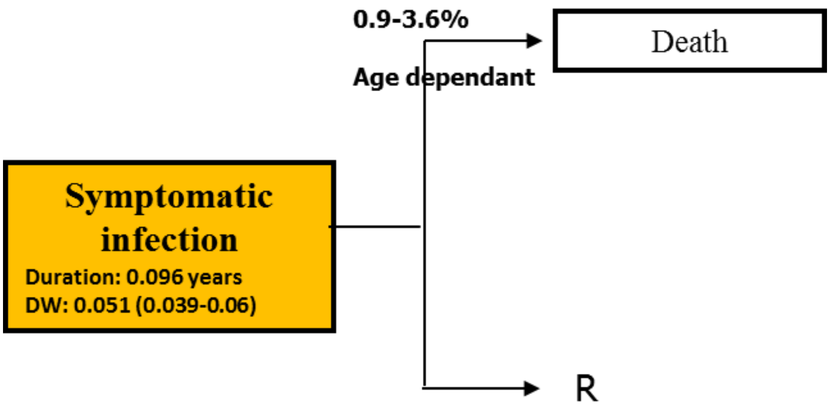

Risk of symptomatic infection and duration of disease

Deep, organ-space and superficial surgical site infections (SSIs) were defined in accordance with the ECDC case definition (ECDC, 2012).

In an attempt to provide an outcome tree reflecting the natural history of healthcare associated SSI, a systematic review of the literature was undertaken. During the initial search strategy, it became clear that it was impossible to obtain a full picture of all attributable deaths, complications, sequelae and length of hospital stay due to the heterogeneous nature of deep, organ-space and superficial SSIs.

We therefore decided to group all SSIs and focus on one sequelae: the proportion of case fatalities. The latter was set to 0.9% for patients aged under 65 years and to 3.6% for patients 65 years and over (Astagneau, 2001). However, this approach underestimated the burden of healthcare-associated SSI which would need to include long-term sequelae, such as mobility restrictions and reoperations following joint replacement surgery.

Model input summary

Table 1. Transition probabilities used in the outcome tree

Health outcome (Health state)	Distribution of health states in health outcome	Transition probability	Source/assumption
Deaths following symptomatic infection		0.9% < 65<br>3.6% >= 65	Astagneau P, 2001

Table 2. Disability weights and duration

Health outcome (Health state)	Disability Weight (DW) (Haagsma, 2015)		Duration	
	DW	Label	In years	Source/assumption
Symptomatic disease	0.051 (0.039-0.06)	Infectious disease, acute episode, moderate	0.096	Coskun, 2005

References

Astagneau P, Rioux C, Golliot F, Brucker G, Group INS. Morbidity and mortality associated with surgical site infections: results from the 1997- 1999 INCISO surveillance. J Hosp Infect. 2001 Aug;48(4):267-74.

Coskun, D., Aytac, J., Aydinli, A., & Bayer, A. (2005). Mortality rate, length of stay and extra cost of sternal surgical site infections following coronary artery bypass grafting in a private medical centre in Turkey. J Hosp Infect, 60(2), 176-179.

European Centre for Disease Prevention and Control. Point prevalence survey of healthcare- associated infections and antimicrobial use in European acute care hospitals – protocol version 4.3. Stockholm: ECDC; 2012.

Haagsma JA, Maertens de Noordhout C, Polinder S, Vos T, Havelaar AH, Cassini A, Devleesschauwer B, Kretzschmar ME, Speybroeck N, Salomon JA. Assessing disability weights based on the responses of 30,660 people from four European countries. Population Health Metrics 2015; 13: 10

Healthcare-associated urinary tract infection (HA UTI)

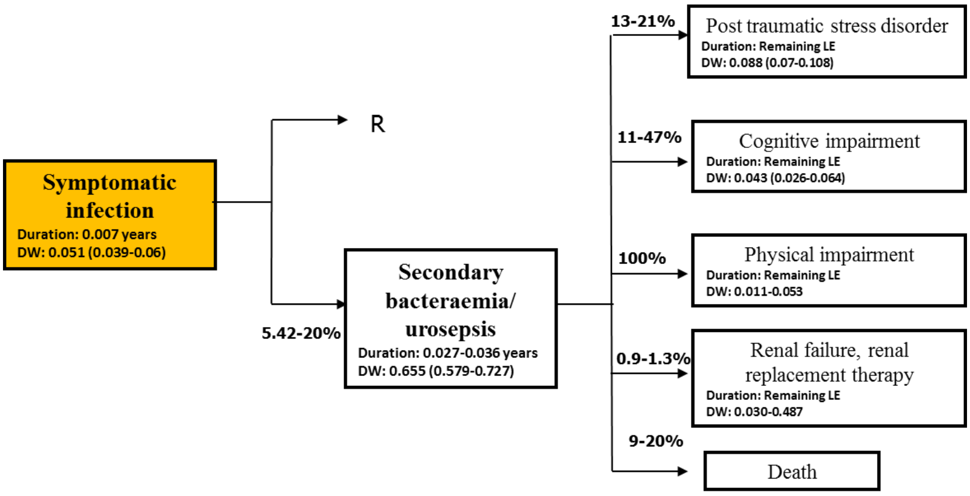

Risk of symptomatic infection and duration of disease

Healthcare-associated urinary tract infection (HA UTI) is defined in accordance with the ECDC case definition (ECDC, 2012). A systematic review of the literature was performed to estimate the sequelae and the probability of developing these sequelae following a UTI. This review took into consideration the role of co-morbidities by estimating the attributable impact of a UTI: attributable mortality, attributable risk of developing sequelae and duration (effect on length of hospital stay).

The extracted literature was limited to catheter-associated UTIs because no specific literature was found for more general search terms.

Attributable ICU and hospital mortality due to UTIs was not statistically significant and we therefore did not attribute a case-fatality rate to UTIs directly. The attributable length of stay for patients with urinary tract infections was 2.6 days (Chant, 2011).

According to the ECDC surveillance definition for UTI used in the point prevalence study, which was the source of the incidence data, both the presence of bacteruria and symptoms are required for the diagnosis of HA UTI. From the systematic literature review, we could not find evidence of the risk of secondary bacteraemia or urosepsis among patients with symptomatic HA UTI, in order to calculate the transitional probability related to complications and death. Therefore we indirectly estimated this risk by combining the risk of developing secondary bacteraemia/urosepsis in critically-ill patients with catheter-associated bacteruria, 1.3–4.8% (Laupland, 2002; Laupland, 2005; Clec'h, 2007), and the risk of developing a urinary tract infection in patients with bacteruria, 24% (Saint, 2000). The resulting transitional probability was set to 5.42–20%.

We assumed that all patients developing bacteraemia/urosepsis will have the same outcomes and relative risks as those developing sepsis. For details, please refer to the bloodstream infection outcome tree.

Model input summary

Table 1. Transition probabilities used in the outcome tree

Health outcome (Health state)	Distribution of health states in health outcome	Transition probability	Source/assumption
Secondary bacteraemia/urosepsis		5.42-20%	Laupland, 2002; Laupland, 2005; Clec'h , 2007; Saint, 2000
Post-traumatic stress disorder (PTSD) following severe sepsis or septic shock		13-21%	Kessler, 1995; Deja, 2006; Schelling, 1998; Stoll, 1999; Kapfhammer, 2004; Hopkins, 2005
Cognitive impairment following severe sepsis or septic shock		11-47%	Hopkins, 2005; Iwashyna, 2010
Physical impairment following severe sepsis or septic shock		100%	Hopkins, 2005; Herridge, 2003; Hofhuis, 2008
Renal failure and renal replacement therapy following severe sepsis or septic		0.9-1.3%	Gallagher, 2014; Wisplinghoff, 2004

shock			
Deaths following secondary bacteraemia/urosepsis		9-20%	Renaud, 2001; Olaechea, 2013

Table 2. Disability weights and duration

Health outcome (Health state)	Disability Weight (DW) (Haagsma, 2015)		Duration	
	DW	Label	In years	Source/assumption
Symptomatic disease	0.051 (0.039-0.06)	Infectious disease, acute episode, moderate	0.007	Chant, 2011
Secondary bacteraemia/urosepsis	0.655 (0.579-0.727)	Intensive care unit admission	0.027-0.036	Olaechea, 2013; Renaud, 2001

Post-traumatic stress disorder (PTSD) following severe sepsis or septic shock	0.088 (0.07-0.108)	Subacute sclerosing panencephalitis – phase 1<br><br>(chosen according to best fitting description)	Remaining life expectancy	
Cognitive impairment following severe sepsis or septic shock	0.043 (0.026-0.064)	Intellectual disability, mild	Remaining life expectancy	
Physical impairment following severe sepsis or septic shock	0.011-0.053	Range between Motor impairment, mild and Motor impairment, moderate	Remaining life expectancy	
Renal failure and renal replacement therapy following severe sepsis or septic shock	0.487-0.030	Range between End-stage renal disease, on dialysis and End-stage renal disease, with kidney transplant	Remaining life expectancy	

References

Chant, O. M. Smith, J. C. Marshall, and J. O. Friedrich, "Relationship of catheter-associated urinary tract infection to mortality and length of stay in critically ill patients: a systematic review and meta-analysis of observational studies," Critical Care Medicine, vol. 39, no. 5, pp. 1167–1173, 2011

Clec'h, C., Schwebel, C., Francais, A., Toledano, D., Fosse, J. P., Garrouste-Orgeas, M., OutcomeRea Study, G. (2007). Does catheter-associated urinary tract infection increase mortality in critically ill patients? Infect Control Hosp Epidemiol, 28(12), 1367-1373

Deja, M., denke, C., Weber-carstens, S., Schroder, J., Pille, C. E., Hokema, F., Falke, k. J. & Kaisers, U. 2006. Social support during intensive care unit stay might improve mental impairment and consequently health-related quality of life in survivors of severe acute respiratory distress syndrome. Crit Care, 10, R147.

Eidelman LA, Putterman D, Putterman C, Sprung CL. The spectrum of septic encephalopathy. Definitions, etiologies, and mortalities. JAMA. 1996;275(6):470-3.

Engel C, Brunkhorst FM, Bone HG, Brunkhorst R, Gerlach H, Grond S, et al. Epidemiology of sepsis in Germany: results from a national prospective multicenter study. Intensive Care Med. 2007;33(4):606-18.

European Centre for Disease Prevention and Control. Point prevalence survey of healthcare-associated infections and antimicrobial use in European acute care hospitals – protocol version 4.3. Stockholm: ECDC; 2012.

Gallagher M, Cass A, Bellomo R, Finfer S, Gattas D, Lee J, et al. Long-Term Survival and Dialysis Dependency Following Acute Kidney Injury in Intensive Care: Extended Follow-up of a Randomized Controlled Trial. PLoS Med. 2014;11(2):e1001601

Garnacho-montero, J., Madrazo-osuna, J., Garcia-garmendia, J. L., ortiz-leyba, C., Jimenez-jimenez, F. J., Barrero-almodovar, A., garnacho- montero, M. C. & Moyano-del-estad, M. R. 2001. Critical illness polyneuropathy: risk factors and clinical consequences. A cohort study in septic patients. Intensive Care Med, 27, 1288-96.

Haagsma JA, Maertens de Noordhout C, Polinder S, Vos T, Havelaar AH, Cassini A, Devleesschauwer B, Kretzschmar ME, Speybroeck N, Salomon JA. Assessing disability weights based on the responses of 30,660 people from four European countries. Population Health Metrics 2015; 13: 10

Herridge, m. S., Cheung, A. M., Tansey, C. M., Matte-martyn, A., Diaz-granados, N., al-saidi, F., Cooper, A. B., Guest, C. B., Mazer, C. D., Mehta, S., Stewart, T. e., Barr, A., Cook, D. & Slutsky, A. S. 2003. One-Year Outcomes in Survivors of the Acute Respiratory Distress Syndrome. New England Journal of Medicine, 348, 683-693.

Hofhuis, J. g., Spronk, P. E., Van Stel, H. F., Schrijvers, A. J., Rommes, J. H. & bakker, J. 2008. The impact of severe sepsis on health-related quality of life: a long-term follow-up study. Anesth Analg, 107, 1957-64.

Hopkins, R. o., Weaver, L. K., Collingridge, D., Parkinson, R. B., Chan, K. J. & Orme, j. F., Jr. 2005. Two-year cognitive, emotional, and quality-of- life outcomes in acute respiratory distress syndrome. Am J Respir Crit Care Med, 171, 340-7

Iwashyna, t. J., Ely, E. W., Smith, D. M. & Langa, K. M. 2010. Long-term cognitive impairment and functional disability among survivors of severe sepsis. JAMA, 304, 1787-94.

Kapfhammer, h. P., Rothenhausler, H. B., Krauseneck, T., Stoll, C. & Schelling, G. 2004. Posttraumatic stress disorder and health-related quality of life in long-term survivors of acute respiratory distress syndrome. Am J Psychiatry, 161, 45-52.

Khan, J., harrison, T. B., Rich, M. M. & Moss, M. 2006. Early development of critical illness myopathy and neuropathy in patients with severe sepsis. Neurology, 67, 1421-5.

Laupland, K. B., Bagshaw, S. M., Gregson, D. B., Kirkpatrick, A. W., Ross, T., & Church, D. L. (2005). Intensive care unit-acquired urinary tract infections in a regional critical care system. Crit Care, 9(2), R60-65.

Laupland, k. B., Zygun, D. A., Davies, H. D., Church, D. L., Louie, T. J. & Doig, C. j. 2002. Incidence and risk factors for acquiring nosocomial urinary tract infection in the critically ill. J Crit Care, 17, 50-7.

Olaechea PM, Palomar M, Alvarez-Lerma F, Otal JJ, Insausti J, Lopez-Pueyo MJ, et al. Morbidity and mortality associated with primary and catheter-related bloodstream infections in critically ill patients. Rev Esp Quimioter. 2013;26(1):21-9.

Oppert M, Engel C, Brunkhorst FM, Bogatsch H, Reinhart K, Frei U, et al. Acute renal failure in patients with severe sepsis and septic shock--a significant independent risk factor for mortality: results from the German Prevalence Study. Nephrol Dial Transplant. 2008;23(3):904-9.

Pittet D, Tarara D, Wenzel RP. Nosocomial bloodstream infection in critically ill patients. Excess length of stay, extra costs, and attributable mortality. JAMA : the journal of the American Medical Association. 1994;271(20):1598-601.

Rangel-Frausto MS, Pittet D, Costigan M, Hwang T, Davis CS, Wenzel RP. The natural history of the systemic inflammatory response syndrome (SIRS). A prospective study. Jama. 1995;273(2):117-23.

Renaud B, Brun-Buisson C, Group IC-BS. Outcomes of primary and catheter-related bacteremia. A cohort and case-control study in critically ill patients. Am J Respir Crit Care Med. 2001;163(7):1584-90.

Saint, S. (2000). Clinical and economic consequences of nosocomial catheter-related bacteriuria. American Journal of Infection Control, 28(1), 68–75

Schelling, g., Stoll, C., Haller, M., Briegel, J., Manert, W., Hummel, T., Lenhart, A., heyduck, M., Polasek, J., Meier, M., Preuss, U., Bullinger, M., Schuffel, W. & Peter, K. 1998. Health-related quality of life and posttraumatic stress disorder in survivors of the acute respiratory distress syndrome. Crit Care Med, 26, 651-9.

Stoll, B. j., Hansen, N. I., Adams-chapman, I., Fanaroff, A. A., Hintz, S. R., Vohr, B. & Higgins, R. D. 2004. Neurodevelopmental and growth impairment among extremely low-birth-weight infants with neonatal infection. Jama, 292, 2357-65.

Wisplinghoff H, Bischoff T, Tallent SM, Seifert H, Wenzel RP, Edmond MB. Nosocomial bloodstream infections in US hospitals: analysis of 24,179 cases from a prospective nationwide surveillance study. Clinical infectious diseases : an official publication of the Infectious Diseases Society of America. 2004;39(3):309-17.
